# Active Surveillance for COVID-19 Vaccine Safety Using Sequential Analysis in Korea: Population-Based Retrospective Observational Study

**DOI:** 10.2196/75094

**Published:** 2026-04-23

**Authors:** Na-Young Jeong, Haerin Cho, Heehyun Won, Suvin Park, Joongyub Lee, Hyesook Park, Nam-Kyong Choi

**Affiliations:** 1 Health Science Convergence Research Institute, College of Science & Industry Convergence, Ewha Womans University, Seoul, Republic of Korea; 2 Department of Health Convergence, College of Science & Industry Convergence, Ewha Womans University, 52 Ewhayeodae-gil, Seodaemun-gu, Seoul, 03760, Republic of Korea, 82 2-3277-6585, 82 2-3277-2867; 3 Department of Preventive Medicine, Seoul National University College of Medicine, Seoul, Republic of Korea; 4 Department of Preventive Medicine, College of Medicine, Ewha Womans University, Seoul, Republic of Korea; 5 Graduate School of Industrial Pharmaceutical Science, College of Pharmacy, Ewha Womans University, Seoul, Republic of Korea

**Keywords:** vaccines, safety, active surveillance, near real-time monitoring, COVID-19 vaccine

## Abstract

**Background:**

With the advent of new vaccines, including the COVID-19 vaccines introduced during the recent pandemic, the need for near real-time active surveillance has increased to support timely regulatory decision-making.

**Objective:**

This study aimed to assess the feasibility of sequential monitoring for potential adverse events following immunization in Korea, focusing on COVID-19 vaccines.

**Methods:**

This population-based study used a linked database that combined the COVID-19 registry with national health insurance claims data. Participants included individuals older than 12 years who received either monovalent or bivalent COVID-19 vaccines in Korea between February 2021 and March 2023. Monthly retrospective sequential testing was performed for 3 prespecified outcomes (acute myocardial infarction, myocarditis, and anaphylaxis) as well as a negative control event (colonic diverticulitis). A Poisson-based maximized sequential probability ratio test was applied to compare postvaccination incidence rates with historical background rates, accounting for multiple testing and claims processing delays. Analyses were stratified by age group, vaccine platform, and dose.

**Results:**

This study included over 43 million monovalent and 6.3 million bivalent vaccine recipients. Sequential analyses identified statistical signals for myocarditis following mRNA vaccines in individuals aged 12 to 64 years and protein subunit vaccines in those aged 40 to 64 years. Signals for anaphylaxis were observed following mRNA and nonreplicating viral vector vaccines in individuals older than 18 years. No safety signals were identified for acute myocardial infarction or colonic diverticulitis. Sequential monitoring detected signals for myocarditis and anaphylaxis before regulatory authorities took safety actions, with the earliest signals observed on September 30, 2021, and April 30, 2021, respectively.

**Conclusions:**

Near real-time sequential testing detected statistical safety signals for myocarditis and anaphylaxis following COVID-19 vaccination. These signals were recognized by the regulatory authority as being associated with the vaccines, demonstrating the potential of this approach to detect signals requiring further causality assessments, particularly for newly introduced vaccines at an early stage.

## Introduction

Active surveillance for vaccine safety is an ongoing and systematic process designed to comprehensively identify adverse events (AEs) in a population [1,2]. This approach plays a critical role in maintaining trust in vaccines by promptly providing data to evaluate potential associations between vaccinations and AEs, thereby supporting regulatory decision-making [3]. Active surveillance can be broadly classified into two types: (1) near real-time (NRT) sequential monitoring using electronic health records (EHRs) or claims databases generated during routine health care services, and (2) cohort event monitoring, which collects primary data through individual surveys to assess postvaccination experiences [4]. NRT sequential monitoring is particularly effective for the early detection of vaccine safety signals when a new vaccine is introduced, a vaccine brand is changed, or the recommended target population is expanded [5]. Sequential monitoring has been widely conducted in various countries, including the United States and European nations, to evaluate the safety of various vaccines.

In the United States, NRT sequential monitoring is primarily performed by the Vaccine Safety Datalink (VSD) [1,6] under the Centers for Disease Control and Prevention (CDC) and by the Biologics Effectiveness and Safety (BEST) Initiative [7] under the Food and Drug Administration (FDA). Since the mid-2000s, the VSD has conducted rapid cycle analysis using weekly updated data files [8], and the results have been published for several vaccines, including seasonal influenza [9-11], H1N1 influenza [12], rotavirus [13], human papillomavirus [14,15], pneumococcal conjugate [16], and meningococcal conjugate vaccines [8]. More recently, sequential analyses have been applied to studies on COVID-19 vaccines to rapidly generate safety evidence following vaccine rollout [17-19]. Similarly, the post-licensure rapid immunization safety monitoring system [20], part of the FDA Sentinel Initiative, initially conducted sequential monitoring for influenza vaccines [21]. Subsequently, the BEST Initiative has employed sequential testing methods for active COVID-19 vaccine safety surveillance [22-24].

In Europe, the Accelerated Development of Vaccine Benefit-Risk Collaboration in Europe, a public-private partnership, assessed the feasibility of NRT monitoring for vaccine coverage, benefits, and risks [5]. Building on the methodologies and tools developed by the Accelerated Development of Vaccine Benefit-Risk Collaboration in Europe, the Vaccine Monitoring Collaboration for Europe leveraged EHRs from various European countries to actively monitor the postmarketing safety and effectiveness of COVID-19 vaccines [25]. Additionally, studies have evaluated the feasibility of sequential monitoring using national datasets, such as the Clinical Practice Research Datalink in the United Kingdom [26,27]. These systems facilitate safety monitoring during the early stages of new vaccine introductions and support ongoing monitoring of annual vaccination programs, thereby reinforcing public trust in vaccination.

Early detection of potential safety signals through NRT sequential monitoring enables timely decisions regarding the need for further comprehensive analyses. By using longitudinal observational data, such as EHRs or health insurance claims data, sequential monitoring comprehensively identifies safety signals in real-world populations, including those excluded from clinical trials [28]. In South Korea, studies using nationwide health insurance claims data have demonstrated the potential to detect postmarketing safety signals [29,30]. However, the feasibility of NRT monitoring of vaccine safety using these data has not yet been assessed. Furthermore, prior studies have not used real-world data to investigate whether sequential analysis can accurately identify potential AEs requiring further rigorous epidemiological assessment, or how quickly such events can be detected.

During the COVID-19 pandemic, the importance of early detection of safety signals became even more apparent due to limited preapproval safety evidence on vaccines. NRT sequential monitoring is particularly valuable for identifying risks when new vaccines are introduced, vaccine types or brands are updated, or vaccination eligibility expands. The results of such monitoring can help prioritize new epidemiological studies or inform regulatory actions. Given the ongoing development of various new vaccines, the need for NRT safety monitoring continues to grow. Therefore, this study aims to assess the feasibility of applying sequential testing methods using a nationwide linked database in South Korea. We conducted an exploratory analysis using recently introduced COVID-19 vaccination data. Additionally, the results of this sequential analysis were compared with known safety evidence and the timing of regulatory actions related to COVID-19 vaccine safety in Korea.

## Methods

### Settings and Study Population

This study used a nationwide database combining the COVID-19 registry from the Korea Disease Control and Prevention Agency (KDCA) and the health insurance claims data from the National Health Insurance Service (NHIS). Korea has a universal health insurance system that provides coverage for the entire population. Claims data are generated when health care providers deliver services to patients and subsequently file claims with the NHIS. The NHIS compiles a comprehensive database that includes information on insurance eligibility, premiums, medical services, and health care institutions [31]. This database encompasses records of inpatient, outpatient, and emergency department visits. Since the launch of the national COVID-19 vaccination program on February 26, 2021, the KDCA has managed vaccination records for the entire population [32]. The linked database includes information on COVID-19 vaccinations, laboratory-confirmed SARS-CoV-2 infections, diagnoses, prescribed medications (eg, drug codes, daily dosage, and duration), and health care procedures. It also contains timestamps for data recording, enabling us to track the dates when the data were accrued.

For this study, we used the KDCA COVID-19 registry data from February 26, 2021, to June 30, 2023, and NHIS health insurance claims data from January 1, 2017, to June 30, 2023. The study population consisted of individuals aged 12 years and older who received either monovalent or bivalent COVID-19 vaccines in Korea. Only individuals with Korean nationality who were enrolled in the national health insurance during the study period were included. Individuals participating in clinical trials for COVID-19 vaccines, those vaccinated abroad, or those with erroneous or duplicate vaccination records were excluded.

### Exposure

The exposures of interest included all COVID-19 monovalent vaccines used for primary doses (ie, first and second doses) and booster doses, as well as bivalent COVID-19 vaccines administered in Korea. Korea introduced 6 types of COVID-19 monovalent vaccines, including those based on mRNA, nonreplicating viral vector, and recombinant protein platforms (Table S1 in Multimedia Appendix 1). Our analysis focused on all primary and booster doses of monovalent COVID-19 vaccines administered from February 26, 2021, to September 30, 2022. During the 2022-2023 winter season, 4 types of mRNA bivalent vaccines were introduced in Korea (Table S2 in Multimedia Appendix 1). As part of the COVID-19 winter booster vaccination program, 1 dose of a bivalent vaccine was recommended regardless of the specific vaccine type. This study included all bivalent COVID-19 vaccines administered from October 11, 2022, to March 31, 2023.

To account for vaccine platforms and recommended dose schedules, we categorized the exposure groups based on the type of vaccines and dose number: mRNA monovalent vaccine (doses 1, 2, and 3), non-replicating viral vector monovalent vaccine (doses 1 and 2), recombinant protein–based monovalent vaccine (doses 1, 2, and 3), and mRNA bivalent vaccine doses. All analyses were stratified by platform and dose number.

### Outcomes

This study selected outcomes of interest by considering both the associations between COVID-19 vaccines and AEs, and the timing of regulatory actions. The selection process involved reviewing the priority lists of adverse events of special interest (AESIs) for COVID-19 vaccines published by the Safety Platform for Emergency Vaccines [33], the VSD [17], and the BEST Initiative [34]. We also considered a list of AEs deemed causally or suspiciously related to COVID-19 vaccines by the KDCA (Table S3 in Multimedia Appendix 1). Through this process, 3 conditions were chosen as the outcomes of interest based on different causal association scenarios: anaphylaxis, myocarditis, and acute myocardial infarction (AMI). Each of these conditions was commonly listed as AESIs by the Safety Platform for Emergency Vaccines, VSD, and BEST Initiative. Anaphylaxis was considered an AESI that was causally associated with COVID-19 vaccines by the KDCA even before vaccine introduction, given that it can potentially be induced by all vaccine types [35]. Subsequent literature further supported this association [36-38]. Myocarditis was included in the AESI lists and, following the vaccine rollout, causal associations were recognized for mRNA and NVX-CoV2373 vaccines [39]. AMI, initially listed as an AESI, has not shown a definitive causal relationship with vaccination in postvaccination epidemiological studies.

In addition, a negative control event was selected—an event for which there is no evidence of a causal association with vaccination and that has not been flagged as a signal [40]. Colonic diverticulitis was chosen as the negative control event, as it was not included in COVID-19 AESI lists and is generally considered unrelated to vaccination. Moreover, this acute condition typically requires immediate treatment, making it unlikely to show significant variations in health care use between the prepandemic and pandemic periods [41].

Table S4 in Multimedia Appendix 1 presents the operational definitions for the selected AESIs and the negative control event. To maintain sensitivity and enable timely analysis for NRT sequential monitoring, operational definitions were based solely on diagnostic codes. The diagnostic codes and the lengths of risk periods were determined by referencing definitions employed by VSD [17] and BEST [34], as well as expert opinions from clinicians in the relevant medical fields.

### Study Design

This study conducted monthly sequential testing to compare the background incidence rates of outcomes of interest with the incidence rates observed during the risk period following COVID-19 vaccination. The study design consisted of three steps: (1) estimation of background incidence rates for the outcomes of interest, (2) analysis of their incidence rates during the postvaccination risk periods, and (3) application of the sequential testing method. Sensitivity analyses were performed when a signal met the statistical threshold.

Sequential testing, which adjusts for multiple comparisons through repeated analyses over time, can rapidly identify potential safety concerns [1,42]. It generates a statistical signal as soon as there is sufficient evidence to reject the null hypothesis of no excess risk, thereby providing timely safety information that can enhance public confidence in vaccines [42]. Sequential testing methods are categorized into continuous sequential methods, which analyze data frequently (eg, weekly or monthly), and group sequential methods, which analyze data at discrete intervals after a specific amount of data has been accumulated [43]. This study adopted a continuous sequential method, known for its superior performance in using data aggregated over short intervals [44]. Among the continuous sequential methods, the maximized sequential probability ratio test (MaxSPRT), developed by VSD for sequential monitoring, is particularly suited for ongoing surveillance as it continues monitoring until the statistical threshold is reached or the maximum sample size is attained [44]. This study used Poisson MaxSPRT (PMaxSPRT) to compare the observed number of cases of AEs with the expected number derived from historical controls, especially for rare events during a large-scale vaccination campaign.

### Statistical Analysis

#### Calculation of Background Rates for AESIs

Background incidence rates for AESIs, serving as historical controls, were estimated from the 2018 and 2019 incidence data. The prepandemic period was selected to avoid potential pandemic-related changes in health care use during the early phase of COVID-19.

The cohort for calculating background rates was defined as individuals with national health insurance eligibility as of January of each respective year. The cohort entry date was set as the later of either January 1 of the respective year or the date when the individual met the clean period criteria. The clean period was defined as the interval preceding cohort entry during which the AESI must not have occurred, serving as a requirement for inclusion in the study cohort. The specific duration varied depending on each AESI and negative control event. Person-time at risk for each individual was calculated from the cohort entry date to the earliest of the following: the date of occurrence of the event, December 31 of the same year, or the date of death. If follow-up ended due to an event, individuals could re-enter the cohort if they subsequently met the clean period criteria (Figure S1 in Multimedia Appendix 1).

For each AESI, the annual incidence rates per 100,000 person-years with exact Poisson 95% CIs were calculated by age group. To enhance the sensitivity of the analysis, the lower annual incidence rate between 2018 and 2019 was used as the background rate for each AESI and age group. Monthly incidence rates were also calculated for each year to assess potential seasonality. This approach allowed for sensitivity analysis by using the average monthly background rate for the corresponding months in each sequential test.

#### Analysis of Incidence Rates During the Risk Period

Although this study retrospectively analyzed data, it was designed under the assumption of conducting sequential analyses starting from the initiation of COVID-19 vaccination. Considering that health care claims from medical care institutions are typically submitted on a weekly or monthly basis, the analysis frequency was set to monthly intervals. Analyses were conducted using data accrued to the database by the end of each month, starting from March 31, 2021. For eligible individuals exposed at each sequential analysis point, the incidence rates of AESIs during the risk period were calculated by age group, vaccine platform, and dose. Follow-up ended upon death or the analysis date, if it occurred before the risk period concluded (Figure S2 in Multimedia Appendix 1).

The follow-up period accounted for delays between the date of medical care and data accrual, which may vary by condition, clinical setting, and institution type. Delays in data accrual during the study period were evaluated using patterns from the prepandemic (January 2019-December 2019) and early pandemic (February 2020-June 2020) periods. For each AESI, monthly accrual rates of claims associated with diagnoses in a given month were calculated, and average delays were derived by analyzing their distribution across subsequent months. The period with higher accrual rates was used to minimize false signal detection. Claims were considered fully accrued once 95% of claims were collected following the month of diagnosis, and the entire follow-up period was used thereafter.

Although vaccination records may also be subject to data delays, they were not considered in this study, as individuals were followed from the recorded exposure date, minimizing the impact on the results. Figure S3 in Multimedia Appendix 1 illustrates the diagram for calculating the follow-up period using historical data on accrual delays.

Sequential analyses incorporated updated data at each testing point. Cases excluded in earlier analyses due to delays in data accrual could be included in subsequent analyses. Similarly, cases initially classified as incident AESIs could be reclassified as having a prior history based on the updated data.

#### Implementation of the Sequential Testing Method

Sequential analyses were conducted monthly following the initiation of the COVID-19 vaccination program, using the PMaxSPRT method. At each analysis point, the number of observed events during the post-vaccination risk period was compared to the expected number of events calculated based on background rates. The expected number of events for individuals vaccinated between month *t* and *s* was calculated by multiplying the number of person-days (*A*) during the risk period (from *w* d to *T_i_* d for each individual i) by the background rate (*θ*) and adjusting for data delay by incorporating *P*(*s,t*). To account for reporting delays arising from partially accrued data, we introduced an adjustment factor *P*(*s,t*), defined as the proportion of AESIs that occurred in study month and were observed by month *s* [34]. The process used to adjust for data accrual delays is illustrated in Figure S4 in Multimedia Appendix 1.


(1) $$\sum_{t=1}^{s}\sum_{i=1}^{n_{t}}\sum_{w=0}^{T_{i}}A_{stiw}\times \theta \times P(s,t)$$

Sequential analyses began on March 31, 2021, or when at least 1 cumulative case was observed within the follow-up period. Data accumulated by the last day of each month were analyzed. The relative risk (RR) and log-likelihood ratio were calculated to compare the likelihood of the observed RR to the null hypothesis. A statistical signal was declared if the log-likelihood ratio exceeded a prespecified critical value. Surveillance ended when one of the following occurred: (1) the statistical signal threshold was met, (2) observed events equaled expected events under the null hypothesis, or (3) the study period ended [42]. Expected event counts under the null hypothesis were estimated using the expected number of vaccinated individuals in the recommended age group within the resident population.

The null hypothesis (*H_0_*: RR≤m) was tested using a one-tailed design with a significance level of 0.05. At least 2 cases were required to declare a signal. Threshold m values were determined based on severity and background incidence of each condition, proposed by Lloyd et al [22] (Table S5 in Multimedia Appendix 1). For colonic diverticulitis, m was set equal to that of anaphylaxis, a severe condition with a comparable background incidence that generally requires hospitalization or emergency department visits. Sensitivity analyses were conducted for all age groups, vaccine platforms, or doses that met the statistical signal threshold, adjusting for time-varying confounders such as seasonality. For outcomes that met the statistical threshold in sequential testing, the timing of the signal was compared with the timing and rationale behind safety measures implemented by the regulatory authority in Korea.

Analyses were performed using SAS Enterprise Guide version 7.1 (SAS Institute, Inc), R version 4.3.1 (R Foundation for Statistical Computing), and the R package *Sequential* version 4.3.3.

### Ethical Considerations

This study was approved by the institutional review board of Ewha Womans University (ewha-202404-0014-01). Informed consent was not required due to the utilization of anonymized patient data.

## Results

### Baseline Characteristics

Table 1 summarizes the sociodemographic and vaccination characteristics of the Korean population who received COVID-19 vaccines during the study period, stratified by vaccine platform and dose number. Over 43 million individuals received monovalent vaccines, with mRNA vaccines being the most frequently administered across all dose levels. Among individuals aged 65 years and older, ChAdOx1 was the vaccine most commonly administered. Since NVV was recommended only for those aged 18 years or older, most individuals aged 12 to 17 years received other vaccine platforms. Recombinant protein–based vaccines, introduced later than other platforms, had fewer recipients. Nonreplicating viral vector vaccines for booster doses were excluded from sequential monitoring as they were not recommended for booster doses.

**Table 1.** Demographic and vaccination characteristics of COVID-19 vaccine recipients.

Characteristics		Type of vaccine platform					
		mRNA vaccine		Nonreplicating viral vector vaccine		Recombinant protein–based vaccine	
Monovalent vaccine (dose 1)							
Total, n (%)		30,772,595 (100)		12,116,724 (100)		126,269 (100)	
Sex, n (%)							
Male		15,173,824 (49.3)		6,220,454 (51.3)		55,755 (44.2)	
Female		15,598,771 (50.7)		5,896,270 (48.7)		70,514 (55.8)	
Age (y)							
Mean (SD)		42.8 (18.8)		59.0 (12.9)		46.9 (17.5)	
12‐17, n (%)		2,282,889 (7.4)		1 (0)		320 (0.3)	
18‐39, n (%)		11,365,058 (36.9)		1,544,309 (12.7)		51,105 (40.5)	
40‐64, n (%)		13,588,828 (44.2)		5,803,270 (47.9)		53,390 (42.3)	
≥65, n (%)		3,535,820 (11.5)		4,769,144 (39.4)		21,454 (16.7)	
Vaccine brand, n (%)							
Ad26.COV2.S		0 (0)		1,255,754 (10.4)		0 (0)	
BNT162b2		24,290,565 (78.9)		0 (0)		0 (0)	
ChAdOx1		0 (0)		10,860,970 (89.6)		0 (0)	
GBP510/AS03		0 (0)		0 (0)		110 (0.1)	
mRNA-1273		6,482,030 (21.1)		0 (0)		0 (0)	
NVX-CoV2373		0 (0)		0 (0)		126,159 (99.9)	
Monovalent vaccine (dose 2)							
Total, n (%)		32,172,217 (100)		9,034,713 (100)		103,456 (100)	
Sex, n (%)							
Male		15,828,203 (49.2)		4,302,525 (47.6)		44,921 (43.4)	
Female		16,344,014 (50.8)		4,732,188 (52.4)		58,535 (56.6)	
Age (y)							
Mean (SD)		43.11 (8.4)		64.1 (9.0)		48.0 (17.5)	
12‐17, n (%)		2,157,466 (6.7)		0 (0)		42 (0)	
18‐39, n (%)		11,698,042 (36.4)		269,704 (3)		39,033 (37.7)	
40‐64, n (%)		14,698,914 (45.7)		4,177,738 (46.2)		45,336 (43.8)	
≥65, n (%)		3,617,795 (11.2)		4,587,271 (50.8)		19,045 (18.4)	
Vaccine brand, n (%)							
Ad26.COV2.S		0 (0)		0 (0)		0 (0)	
BNT162b2		25,849,287 (80.3)		0 (0)		0 (0)	
ChAdOx1		0 (0)		9,034,713 (100)		0 (0)	
GBP510/AS03		0 (0)		0 (0)		0 (0)	
mRNA-1273		6,322,930 (19.7)		0 (0)		0 (0)	
NVX-CoV2373		0 (0)		0 (0)		103,456 (100)	
Monovalent vaccine (dose 3)							
Total, n (%)		31,806,466 (100)		14,917 (100)		97,145 (100)	
Sex, n (%)							
Male		15,753,046 (49.5)		11,189 (75)		44,237 (45.5)	
Female		16,053,420 (50.5)		3728 (25)		52,908 (54.5)	
Age (y)							
Mean (SD)		51.9 (17.7)		45.8 (12.2)		48.6 (16.0)	
12‐17, n (%)		302,753 (1)		0 (0)		107 (0.1)	
18‐39, n (%)		7,762,384 (24.4)		6281 (42.1)		29,347 (30.2)	
40‐64, n (%)		15,847,317 (49.8)		7252 (48.6)		51,861 (53.4)	
≥65, n (%)		7,894,012 (24.8)		1384 (9.3)		15,830 (16.3)	
Vaccine brand, n (%)							
Ad26.COV2.S		0 (0)		14,820 (99.3)		0 (0)	
BNT162b2		21,504,295 (67.6)		0 (0)		0 (0)	
ChAdOx1		0 (0)		97 (0.7)		0 (0)	
GBP510/AS03		0 (0)		0 (0)		56 (0.1)	
mRNA-1273		10,302,171 (32.4)		0 (0)		0 (0)	
NVX-CoV2373		0 (0)		0 (0)		97,089 (99.9)	
Bivalent vaccine							
Total		6,393,809 (100)		—^a^		—	
Sex, n (%)							
Male		3,236,617 (50.6)		—		—	
Female		3,157,192 (49.4)		—		—	
Age (y)							
Mean (SD)		63.3 (17.0)		—		—	
12-17, n (%)		33,629 (0.5)		—		—	
18-39, n (%)		697,503 (10.9)		—		—	
40‐64, n (%)		2,062,883 (32.3)		—		—	
≥65, n (%)		3,599,794 (56.3)		—		—	
Vaccine brand, n (%)							
BNT162b2 BA.1		878,184 (13.7)		—		—	
BNT162b2 BA.4/5		3,393,011 (53.1)		—		—	
mRNA-1273 BA.1		1,910,876 (29.9)		—		—	
mRNA-1273 BA.4/5		211,738 (3.3)		—		—	

a Not applicable.

Approximately 6.3 million individuals received bivalent vaccines, with the majority being aged 65 years or older, reflecting the prioritization of older adults, immunocompromised individuals, and high-risk groups. BNT162b2 BA.4/5 was the most frequently administered bivalent vaccine. Figures S5 and S6 in Multimedia Appendix 1 illustrate the weekly distribution of vaccinations by vaccine type and dose number for both monovalent and bivalent vaccines.

### Calculation of Background Rates for AESIs

Table 2 presents the annual background rates of AESI derived from data for 2018, 2019, and 2020. For most AESI across all age groups, the background rates in 2020 were either statistically significantly lower or similar to those in the pre-COVID-19 period. This study used the lower incidence rate between 2018 and 2019 as the background rate for each AESI and age group.

**Table 2.** Background incidence rates of prespecified adverse events of special interest by age group.

Adverse events of special interest and age group	Pre-COVID-19 pandemic, incidence rates (95% CI)^a^		COVID-19 pandemic (2020^b^), incidence rates (95% CI)
	2018	2019	
Acute myocardial infarction			
Total	108.36 (107.43-109.29)	113.75 (112.81-114.70)	99.29 (98.36-100.23)
12–17 years	4.88 (4.14-5.76)	5.66 (4.85-6.61)	3.86 (3.17-4.70)
18–39 years	13.00 (12.45-13.58)	15.88 (15.26-16.52)	13.04 (12.46-13.66)
40–64 years	96.34 (95.01-97.68)	101.68 (100.32-103.05)	89.67 (88.34-91.02)
≥65 years	386.04 (381.59-390.54)	381.71 (377.44-386.04)	317.96 (313.95-322.03)
Myocarditis			
Total	3.00 (2.84-3.16)	3.50 (3.34-3.67)	2.57 (2.43-2.73)
12–17 years	8.74 (7.73-9.88)	8.44 (7.43-9.58)	3.94 (3.24-4.78)
18–39 years	1.93 (1.73-2.16)	2.75 (2.50-3.03)	1.66 (1.46-1.89)
40–64 years	2.26 (2.07-2.48)	2.68 (2.47-2.91)	1.93 (1.75-2.14)
≥65 years	4.97 (4.50-5.50)	5.32 (4.84-5.85)	5.32 (4.84-5.86)
Anaphylaxis			
Total	37.25 (36.71-37.81)	39.86 (39.30-40.44)	32.28 (31.75-32.81)
12–17 years	23.63 (21.93-25.46)	25.13 (23.36-27.05)	18.06 (16.50-19.78)
18–39 years	24.72 (23.95-25.51)	26.42 (25.62-27.24)	23.94 (23.14-24.77)
40–64 years	45.99 (45.08-46.92)	48.46 (47.53-49.41)	38.15 (37.30-39.03)
≥65 years	44.46 (42.99-45.97)	48.49 (47.00-50.03)	37.28 (35.95-38.66)
Colonic diverticulitis			
Total	44.34 (43.74-44.94)	47.45 (46.83-48.07)	46.02 (45.40-46.66)
12–17 years	6.47 (5.61-7.46)	7.73 (6.78-8.83)	6.48 (5.57-7.54)
18–39 years	31.32 (30.46-32.21)	34.86 (33.94-35.80)	34.05 (33.10-35.03)
40–64 years	51.29 (50.32-52.27)	54.46 (53.47-55.46)	52.55 (51.54-53.58)
≥65 years	66.60 (64.80-68.46)	67.43 (65.66-69.25)	64.34 (62.59-66.14)

a Incidence rate per 100,000 person-years.

b The background incidence rate for 2020 was calculated for individuals with eligibility information as of February of that year, considering the observation period from February 2020 to December 2020.

### Incidence Rates During the Risk Period

Figures S6-S9 in Multimedia Appendix 1 present the distribution of delays in data accrual for claims made before the introduction of COVID-19 vaccination. For all AESIs, comparisons revealed that diagnoses from inpatient settings had faster data accrual than those from combined inpatient and outpatient settings (Figures S7-S10 in Multimedia Appendix 1). Consequently, the study used data delay distributions based on inpatient diagnostic claims. During the COVID-19 pandemic, over 95% of claims data were accrued faster compared to the prepandemic period. Thus, this study used data accrual delay distributions from inpatient diagnoses recorded between February 2020 and June 2020 to calculate the contribution of the risk period to sequential testing (Table S6 in Multimedia Appendix 1).

The person-time at risk and the number of events at each sequential testing point were calculated, and the expected number of events within the risk period was estimated using background rates. These calculations were performed separately for each age group, vaccine platform, and dose at each testing.

### Implementation of the Sequential Monitoring Method

For AMI and colonic diverticulitis, none of the analyses stratified by age group, vaccine platform, or dose met the statistical signal threshold (Tables S7 and S8 in Multimedia Appendix 1).

For myocarditis, the statistical signal threshold was met for mRNA vaccines in the 12‐17 and 18‐39 age groups across all doses and for the first dose in the 40‐64 age group. The threshold was also met for recombinant protein vaccines in the 40‐64 age group for both the first and second doses. Significant results from sequential testing for myocarditis are summarized in Table 3, while the complete results for all age and dose groups are provided in Table S9 in Multimedia Appendix 1.

**Table 3.** Summary of significant sequential testing results for myocarditis.

Vaccine platform and dose		At data cutoff point				At statistical signal detection point				
		Person-days	Events	RR^a^	LLR^b^^c^	Statistical signal	Date	Events	RR	LLR
12‐17 years										
mRNA										
Dose 1		69,097,626	54	3.38	13.83	Yes	January 31, 2022	17	3.79	5.48
Dose 2		70,515,241	54	3.31	13.22	Yes	January 31, 2022	12	6.70	8.65
Dose 3		7,720,439	12	6.70	8.65	Yes	June 30, 2022	8	11.27	9.20
18‐39 years										
mRNA										
Dose 1		349,949,622	146	7.89	124.16	Yes	September 30, 2021	11	6.92	8.20
Dose 2		375,637,912	115	5.79	70.13	Yes	September 30, 2021	7	9.33	6.92
Dose 3		233,868,158	32	2.59	3.99	Yes	February 28, 2022	11	5.21	5.87
40‐64 years										
mRNA										
Dose 1		408,331,078	61	2.41	5.92	Yes	December 31, 2021	47	2.10	2.40
Recombinant protein–based										
Dose 1		1,676,909	3	30.00	6.14	Yes	June 30, 2022	2	22.22	3.53
Dose 2		1,386,915	3	33.33	6.44	Yes	June 30, 2022	2	40.00	6.64

a RR: relative risk.

b LLR: log-likelihood ratio.

c The log-likelihood ratio is artificially set to 0 when the relative risk is less than 1.

For anaphylaxis, the statistical signal threshold was exceeded for mRNA vaccines in the 18‐39 age group across all doses and for the first and second doses in individuals aged 40 years and older. For nonreplicating viral vector vaccines, the threshold was met following the first and second doses in the 18‐39 and 40‐64 age groups, as well as after the first dose in individuals aged 65 years and older. A summary of significant sequential testing results for anaphylaxis is presented in Table 4, and detailed findings across all age and dose groups are available in Table S10 in Multimedia Appendix 1.

**Table 4.** Summary of significant sequential testing results for anaphylaxis.

Vaccine platform and dose		At data cutoff point				At statistical signal detection point				
		Person-days	Events	RR^a^	LLR^b^^c^	Statistical signal	Date	Events	RR	LLR
18‐39 years										
mRNA										
Dose 1		11,355,193	133	17.30	203.72	Yes	June 30, 2021	2	66.67	5.63
Dose 2		11,687,130	44	5.56	25.49	Yes	August 31, 2021	3	25.00	5.62
Dose 3		7,713,760	13	2.49	1.42	Yes	January 31, 2022	4	9.30	3.94
Nonreplicating viral vector										
Dose 1		1,543,045	43	40.95	100.77	Yes	April 30, 2021	9	900.00	48.59
Dose 2		269,520	4	27.32	9.79	Yes	July 31, 2021	2	20.00	3.33
40‐64 years										
mRNA										
Dose 1		13,578,335	105	6.14	68.59	Yes	June 30, 2021	3	42.86	7.16
Dose 2		14,686,963	48	2.59	6.04	Yes	July 31, 2021	2	28.57	4.00
Nonreplicating viral vector										
Dose 1		5,799,204	64	8.76	59.87	Yes	April 30, 2021	3	42.86	7.16
Dose 2		4,173,320	11	2.09	0.55	Yes	July 31, 2021	2	28.57	4.00
≥65 years										
mRNA										
Dose 1		3,147,253	23	5.35	12.69	Yes	July 31, 2021	14	5.69	8.36
Dose 2		3,613,725	15	3.41	3.91	Yes	August 31, 2021	10	3.25	2.34
Nonreplicating viral vector										
Dose 1		4,759,853	16	2.76	2.45	Yes	July 31, 2021	7	3.65	2.10

a RR: relative risk.

b LLR: log-likelihood ratio.

c The log-likelihood ratio is artificially set to 0 when the relative risk is less than 1.

Sensitivity analyses were conducted for myocarditis and anaphylaxis to account for temporal trends in monthly incidence rates. For myocarditis, no differences were found between the sensitivity and primary analyses (Table S11 in Multimedia Appendix 1). For anaphylaxis, however, the sensitivity analysis showed that the signal threshold was no longer exceeded for the second dose of non-replicating viral vector vaccines in the 40‐64 age group or the first dose in individuals aged 65 years and older (Table S12 in Multimedia Appendix 1).

### Comparison of the Timing of Signal Detection and Implementation of Domestic Safety Measures

The timing of statistical signal detection using the sequential monitoring method was compared to the timing of safety measures implemented by the KDCA (Figure S11 in Multimedia Appendix 1).

For myocarditis, the KDCA initially did not recognize an association between COVID-19 vaccines and myocarditis during the early vaccination phase. However, international reports of myocarditis following mRNA COVID-19 vaccination and reviews by global regulatory agencies prompted further investigation [45,46]. Based on domestic causality assessments, the KDCA announced on March 14, 2022, that myocarditis was causally associated with mRNA COVID-19 vaccination. Subsequently, on December 29, 2022, myocarditis was identified as a potential AE related to recombinant protein–based vaccines [39].

In this study, sequential testing detected statistical signals for myocarditis in different age groups after receiving mRNA vaccines. For the 12‐17 and 18‐39 age groups, the signals were detected in January 2022 and September 2021, respectively. In the 40‐64 age group, signals were detected for mRNA vaccines in December 2021 and for recombinant protein–based vaccines in June 2022. The earliest signal was observed on September 30, 2021, for mRNA vaccines in the 18‐39 age group.

For anaphylaxis, the KDCA recognized a causal relationship with COVID-19 vaccination from the beginning of the vaccination program. Sequential testing detected signals for nonreplicating viral vector vaccines in the 18‐39 age group in April 2021 and for mRNA vaccines in June 2021. In the 40‐64 age group, signals for nonreplicating viral vector vaccines were detected in April 2021, while mRNA vaccines reached the threshold in June 2021, after approximately 80,000 doses. Among individuals aged 65 years and older, signals for both nonreplicating viral vector and mRNA vaccines were detected in July 2021. The earliest signal was observed on April 30, 2021, for the first dose of nonreplicating viral vector vaccines in the 18‐39 and 40‐64 age groups.

## Discussion

### Principal Findings

This study conducted an exploratory analysis to evaluate the applicability of NRT surveillance for recently developed COVID-19 vaccines using a sequential testing method. Monthly analyses were performed using PMaxSPRT to compare background rates with postvaccination incidence rates, accounting for data accrual delays. All COVID-19 vaccines introduced in Korea were analyzed by vaccine platform, age group, and dose for 4 outcomes of interest.

The results showed that the statistical signal threshold for myocarditis was met in individuals younger than 64 years after mRNA vaccination and in those aged 40 to 64 years after recombinant protein–based vaccines. For anaphylaxis, the statistical signal threshold was met following mRNA and nonreplicating viral vector vaccines in individuals aged 18 years and older. No statistical signals were detected for AMI or colonic diverticulitis by the end of surveillance.

Comparison with prior studies revealed consistent findings for myocarditis and anaphylaxis. Lloyd et al [22] identified statistical signals for myocarditis, pericarditis, and anaphylaxis following COVID-19 vaccination in the US population aged 12‐64 years. Similarly, studies analyzing BNT162b2 in adolescents aged 12 years and older also detected signals for myocarditis and pericarditis [23,24]. Wong et al [47], studying US adults aged 65 years and older, reported signals for AMI and other outcomes, but sensitivity analyses accounting for time trends and pandemic background rates showed no signals. These findings align with our study, which detected statistical signals for myocarditis and anaphylaxis but not for AMI.

This study compared the timing of statistical signals with the regulatory actions taken by the KDCA. In Korea, the KDCA delegated COVID-19 vaccine safety monitoring to CoVaSC, which made causality assessments prior to regulatory decisions [32]. Anaphylaxis was recognized as causally associated with COVID-19 vaccination early in the campaign. Sequential testing revealed statistical signals for nonreplicating viral vector vaccines in April 2021 and for mRNA vaccines in June 2021. Subsequent epidemiological studies confirmed the association between COVID-19 vaccines and incident anaphylaxis risk [36,39]. For myocarditis, causality was recognized for mRNA vaccines in March 2022 and for recombinant protein–based vaccines in December 2022 [39]. Sequential testing showed that statistical signals were first detected in September 2021 for mRNA vaccines and in June 2022 for recombinant protein–based vaccines. These findings suggest that NRT sequential testing could have facilitated earlier epidemiological evaluations of high-priority conditions using domestic data.

Sequential monitoring conducted early during the COVID-19 vaccination campaign in countries such as the United States demonstrated the rapid detection of safety signals when applying NRT methods. For instance, sequential testing in the US FDA surveillance program identified statistical signals for AMI, pulmonary embolism, disseminated intravascular coagulation, immune thrombocytopenia, myocarditis, and pericarditis after BNT162b2 vaccination. It also detected a signal for anaphylaxis after mRNA-1273 vaccination [22,47]. In a US pediatric population aged 5‐17 years, sequential testing detected signals for myocarditis and pericarditis [24]. Initial VSD analyses in the United States did not detect signals for AESIs [17], but extended studies later identified signals for Guillain-Barré syndrome with Ad26.COV2.S and myocarditis or pericarditis in younger adults after mRNA vaccination [48,49].

Countries with established NRT sequential monitoring systems identified safety concerns relatively early in their vaccination campaigns. In contrast, Korea’s vaccination campaign began later without a sequential monitoring system in place. As a result, regulatory actions largely relied on international findings, such as safety concerns about myocarditis raised abroad before vaccines were administered to Korean adolescents and young adults. This reliance may have influenced diagnosis coding practices. Cases resembling myocarditis might have been coded as myocarditis, which could have impacted studies based on claims data. Our study, which used a health insurance claims database for sequential analysis, may also have been influenced by these coding practices in analyzing myocarditis, one of our outcomes of interest. Such diagnostic awareness and heightened attention following international safety reports may have increased the likelihood of coding myocarditis, potentially inflating the observed signal strength. If an NRT monitoring system had been established before these issues were raised internationally, or if sequential analysis had been conducted globally at the onset of vaccinations, the time required to detect a myocarditis signal in our study might have been longer. In that case, a lower relative risk might have been estimated. However, had sequential monitoring been implemented domestically, safety signals might have been detected earlier, as demonstrated by this study.

To implement NRT monitoring effectively within the country, further exploration of data sources capable of supporting NRT monitoring is needed. In this study, we used health insurance claims data and addressed data latency by calculating delay distributions based on recent historical data. One advantage of using domestic health insurance claims data is that they offer medical information for the entire population, allowing for the surveillance of even rare AEs. However, given the nature of claims data, inherent delays may occur during several stages of data processing. These include the provision of medical services, submission of claims by medical institutions, review procedures, and subsequent reimbursement by the NHIS. This process can take approximately 3 months or, in some cases, more than 6 months before the data becomes available. Therefore, to enhance NRT monitoring in the future, collaboration with sample hospitals might be considered to collect data based on EHRs, instead of claims data, which may accelerate the data accrual process. Integrating multiple data sources, such as NRT hospital data and EHRs, would allow timely validation of diagnostic codes and help mitigate potential diagnostic biases and misclassification. Furthermore, to prevent spurious signal detection driven by heightened diagnostic awareness, such as increased hospital visits and changes in coding practices observed for myocarditis, future surveillance systems should incorporate clinical validation processes. These should include case verification using detailed medical record review or laboratory findings. Linking diagnostic data with laboratory results or chart reviews could help distinguish genuine cases from those arising due to increased diagnostic activity.

### Strengths and Limitations

To our knowledge, this is the first study in Korea to apply NRT sequential monitoring based on a nationwide vaccination registry and health care data. We used a data accrual delay adjustment method tailored to claims data. This approach enabled early safety surveillance shortly after vaccine rollout. However, this study has several limitations. First, while analyses were stratified by age group, vaccine platform, and dose—key factors influencing safety and timing of COVID-19 vaccination—the study did not account for sex differences. This was because vaccine recommendations and rollout timing differed across age groups. These factors were expected to have a greater impact on safety signal detection during the signal detection phase. Considering potential biological and behavioral differences between sexes, future studies should incorporate subgroup analyses by sex to identify potential variations. Second, as this study was designed as a feasibility assessment, the scope was intentionally limited to 3 AESIs and 1 negative control outcome. The selected outcomes were mostly diagnosed in Korea after being reported as AEs in countries that began vaccination earlier. Such international reports may have influenced health care–seeking behavior, potentially leading to overdiagnosis of conditions with similar clinical presentations. For instance, myocarditis cases in Korea were diagnosed predominantly after international reports, where heightened awareness may have contributed to an increase in the diagnosis of related conditions. Future research should expand sequential monitoring to a broader range of AESIs, such as Guillain-Barré syndrome or thrombosis with thrombocytopenia, to evaluate its applicability across diverse clinical presentations. Third, the operational definitions of AESIs relied solely on diagnostic codes for timely signal detection, which may limit diagnostic validity. For AESIs that met the statistical signal threshold in sequential testing, follow-up epidemiological studies with refined operational definitions are necessary to improve diagnostic accuracy. Fourth, the average data accrual rate, calculated from historical data, may not fully reflect actual delays or unexpected changes during the study period. However, this study minimized potential inaccuracies by using recent historical data. Further efforts should be made to continuously update and incorporate monthly accrual rates from the most recent data to enhance accuracy. Finally, although the sequential analysis controlled for repeated testing within each analysis stratum, no adjustment was made for multiple testing across all strata. As a result, the possibility of an inflated type I error rate at the overall study level cannot be entirely ruled out, and the findings should be interpreted as hypothesis-generating rather than confirmatory.

### Conclusions

In conclusion, this study demonstrated that sequential monitoring can be effectively applied using Korean data sources in situations that require active surveillance, such as monitoring newly developed vaccines. Comparisons between signal detection timelines and KDCA regulatory actions indicated that NRT sequential monitoring could have enabled earlier recognition of safety concerns, such as anaphylaxis and myocarditis, thereby supporting more timely regulatory decisions. Future studies should explore alternative data sources, such as EHRs, to incorporate a broader range of clinical information and improve monitoring timeliness.

## Supplementary material

10.2196/75094 Multimedia Appendix 1 Exposure and AESI definitions, study design diagram, sequential testing results, and supporting tables and figures.

## Abbreviations

AE: adverse event
AESI: adverse events of special interest
AMI: acute myocardial infarction
BEST: Biologics Effectiveness and Safety
CDC: Centers for Disease Control and Prevention
EHR: electronic health record
FDA: Food and Drug Administration
KDCA: Korea Disease Control and Prevention Agency
MaxSPRT: maximized sequential probability ratio test
NHIS: National Health Insurance Service
NRT: near real-time
PMaxSPRT: Poisson maximized sequential probability ratio test
RR: relative risk
VSD: Vaccine Safety Datalink
