# Supplementary material for: Active Surveillance for COVID-19 Vaccine Safety Using Sequential Analysis in Korea: Population-Based Retrospective Observational Study
Source: JMIR Public Health Surveill. 2026 Apr 23;12:e75094. doi: 10.2196/75094 (PMC13105434; doi:10.2196/75094)
Supplement: Multimedia Appendix 1 [file publichealth-v12-e75094-s001.docx]

Supplemental Table S1. COVID-19 monovalent vaccines approved in Korea during the study period

| **Type of  COVID-19 vaccine** | **Platform** | **Recommended targets for primary series** | **Criteria for completion of primary series vaccination (Recommended COVID-19 vaccine dosing interval)** |
| --- | --- | --- | --- |
| **Brand name** | **Date of first**  **vaccination in Korea** | **Recommended targets for booster dose** | **Recommended COVID-19 vaccine booster dosing interval** |
| ChAdOx1 | Non-replicating viral vector vaccine | Aged 50 years and over | Two-dose (8–12 weeks) |
| AstraZeneca | February 26, 2021 | - | - |
| BNT162b2 | mRNA vaccine | Aged 12 years and over | Two-dose (21 days) |
| Pfizer-BioNTech | February 27, 2021 | (3^rd^ dose) Aged 12 years and over | (3^rd^ dose) Three months after the second dose |
| Ad26.COV2.S | Non-replicating viral vector vaccine | Aged 18 years and over | Single dose |
| Janssen | June 10, 2021 | (3^rd^ dose) Aged 18 years and over | (3^rd^ dose) Two months after the second dose^a^ |
| mRNA-1273 | RNA vaccine | Aged 30 years and over | Two-dose (28 days) |
| Moderna | June 16, 2021 | (3^rd^ dose) Aged 18 years and over | (3^rd^ dose) Three months after the second dose |
| NVX-CoV2373 | Recombinant protein-based vaccine | Aged 12 years and over | Two-dose (21 days) |
| Novavax | February 14, 2022 | (3^rd^ dose) Aged 12 years and over | (3^rd^ dose) Three months after the second dose |
| GBP510/AS03 | Recombinant protein-based vaccine | Aged 18 years and over | Two-dose (28 days) |
| SK Bioscience | September 5, 2022 | (3^rd^ dose) Aged 18 years and over | (3^rd^ dose) Three months after the second dose |

Abbreviation: mRNA, Messenger Ribonucleic Acid

^a^ Only those who are vaccinated with Ad26.COV2.S can receive booster dose with Ad26.COV2.S.

| **Type of  COVID-19 vaccine** | **Platform** | **Recommended targets** | **Recommended COVID-19 vaccine dosing interval** |
| --- | --- | --- | --- |
| **Brand name** | **Date of first**  **vaccination in Korea** |  |  |
| mRNA-1273 BA.1 | mRNA vaccine | Aged 18 years and over | 90–120 days after the last COVID-19 vaccination date |
| Moderna | October 11, 2022 |  |  |
| BNT162b2 BA.1 | mRNA vaccine | Aged 12 years and over |  |
| Pfizer-BioNTech | November 7, 2022 |  |  |
| BNT162b2 BA.4/5 | mRNA vaccine | Aged 12 years and over |  |
| Pfizer-BioNTech | November 14, 2022 |  |  |
| mRNA-1273 BA.4/5 | mRNA vaccine | Aged 18 years and over |  |
| Moderna | December 26, 2022 |  |  |

Supplemental Table S2. COVID-19 bivalent vaccines approved in Korea during the study period

Abbreviation: mRNA, Messenger Ribonucleic Acid

Supplemental Table S3. Pre-specified list of adverse events of special interest for COVID-19 vaccines and conditions identified as causally related or potentially associated with COVID-19 vaccination

| **Adverse events of special interest** | **SPEAC [1]** | **VSD  [2]** | **BEST [3]** | **Policy decisions on the association between COVID-19 vaccines and AEs in Korea** |
| --- | --- | --- | --- | --- |
| Acute aseptic arthritis | ✓ |  |  |  |
| Acute disseminated encephalomyelitis | ✓ | ✓ |  | [Listed as reportable AEs]  ChAdOx1 (Prior to vaccine introduction) |
| Acute kidney injury | ✓ |  |  |  |
| Acute liver injury | ✓ |  |  |  |
| Acute myocardial infarction | ✓ | ✓ | ✓ |  |
| Acute pancreatitis | ✓ |  |  |  |
| Acute respiratory distress syndrome | ✓ | ✓ |  |  |
| Anaphylaxis | ✓ | ✓ | ✓ | [Acknowledgement of causality]  All type vaccines (Prior to vaccine introduction) |
| Anosmia/ageusia | ✓ |  |  |  |
| Appendicitis |  | ✓ | ✓ |  |
| Aseptic meningitis | ✓ |  |  |  |
| Bell’s Palsy | ✓ | ✓ | ✓ | [Listed as reportable AEs]  mRNA vaccines (Mar 2022); ChAdOx1 (Dec 2022) |
| Cerebral venous sinus thrombosis |  | ✓ |  | [Listed as reportable AEs]  All type vaccines (Prior to vaccine introduction) |
| Chilblains | ✓ |  |  |  |
| Convulsions/seizures |  | ✓ |  |  |
| Deep vein thrombosis |  |  | ✓ |  |
| Disseminated intravascular coagulation |  | ✓ | ✓ |  |
| Encephalitis/myelitis/encephalomyelitis | ✓ | ✓ | ✓ |  |
| Erythema multiforme | ✓ |  |  | [Listed as reportable AEs]  mRNA vaccines (Mar 2022) |
| Generalized convulsion | ✓ |  |  |  |
| Guillain-Barre Syndrome | ✓ | ✓ | ✓ | [Listed as reportable AEs]  All type vaccines (Prior to vaccine introduction) |
| Immune thrombocytopenia |  | ✓ | ✓ | [Listed as reportable AEs]  Non-replicating viral vector vaccines (Prior to vaccine introduction) |
| Kawasaki disease |  | ✓ |  |  |
| Multisystem inflammation syndrome | ✓ | ✓ | ✓ |  |
| Myocarditis | ✓ | ✓ | ✓ | [Acknowledgement of causality]  mRNA vaccines (Mar 2022);  [Listed as reportable AEs]  NVX-CoV2373 (Dec 2022) |
| Narcolepsy |  | ✓ | ✓ |  |
| Pericarditis | ✓ | ✓ | ✓ | [Acknowledgement of causality]  mRNA vaccines (May 2022);  [Listed as reportable AEs]  NVX-CoV2373 (Dec 2022) |
| Pulmonary embolism |  | ✓ | ✓ |  |
| Rhabdomyolysis | ✓ |  |  |  |
| Single organ cutaneous vasculitis | ✓ |  |  |  |
| Stroke (hemorrhagic and ischemic) |  | ✓ | ✓ |  |
| Subacute thyroiditis | ✓ |  |  |  |
| Thrombocytopenia | ✓ |  |  |  |
| Thrombosis with thrombocytopenia syndrome | ✓ | ✓ |  | [Acknowledgement of causality] Non-replicating viral vector vaccines |
| Thrombotic thrombocytopenic purpura |  | ✓ |  |  |
| Transverse myelitis |  | ✓ | ✓ | [Listed as reportable AEs]  Non-replicating viral vector vaccines (Mar 2022);  mRNA vaccines (Feb 2023) |
| Vaccine-associated enhanced disease | ✓ |  |  |  |
| Venous thromboembolism |  | ✓ |  | [Listed as reportable AEs]  Ad26.COV2.S (Mar 2022) |

Abbreviation: SPEAC, Safety Platform for Emergency vACcines; VSD, Vaccine Safety Datalink; BEST, Biologics Effectiveness and Safety Initiative; AE, Adverse Event.

[1] Law B, Pim C. Third update - Priority List of Adverse Events of Special Interest: COVID-19. Zenodo. 2022. doi: 10.5281/zenodo.6669790.

[2] Klein NP, Lewis N, Goddard K, Fireman B, Zerbo O, Hanson KE, et al. Surveillance for Adverse Events After COVID-19 mRNA Vaccination. JAMA. 2021;326(14):1390-1399. doi: 10.1001/jama.2021.15072.

[3] U.S. Food and Drug Administration. Center for Biologics Evaluation and Research Office of Biostatistics and Epidemiology. COVID-19 Vaccine Safety Surveillance: Active Monitoring Master Protocol. 2021. https://bestinitiative.org/wp-content/uploads/2021/02/C19-Vaccine-Safety-Protocol-2021.pdf

| **KCD-8 codes** | **Risk period** | **Exclusion criteria** |
| --- | --- | --- |
|  | **Setting** |  |
| **Acute myocardial infarction [1–3]** | | |
| - I21.* (Acute myocardial infarction) | Days 1–28 | Cases diagnosed with acute myocardial infarction within 365 days prior to the cohort entry date^a^.   - I21.* (Acute myocardial infarction) - I22.* (Subsequent myocardial infarction) - I23.* (Certain current complications following acute myocardial infarction) - I24.* (Other acute ischaemic heart diseases) - I25.* (Chronic ischaemic heart disease) |
|  | Inpatient; Emergency department |  |
| **Myocarditis [1,2,4]** | | |
| - I40.* (Acute myocarditis) - I51.4 (Myocarditis, unspecified) - B33.22 (Viral myocarditis) | Days 1–42 | Cases diagnosed with myocarditis within 365 days prior to the cohort entry date.   - I40.* (Acute myocarditis) - I41.* (Myocarditis in diseases classified elsewhere) - I51.4 (Myocarditis, unspecified) - B33.22 (Viral myocarditis) |
|  | Inpatient; Emergency department |  |
| **Anaphylaxis [1,2,5]** | | |
| - T78.2 (Anaphylactic-shock, unspecified) - T88.6 (Anaphylactic shock due to adverse effect of correct drug or medicament properly administered) | Days 0–1 | Cases diagnosed with anaphylaxis within 30 days prior to the cohort entry date.   - T78.0 (Anaphylactic shock due to adverse food reaction) - T78.2 (Anaphylactic shock, unspecified) - T80.5 (Anaphylactic shock due to serum) - T88.6 (Anaphylactic shock due to adverse effect of correct drug or medicament properly administered) |
|  | Inpatient; Emergency department |  |
| **Colonic** **diverticulitis [1,6]** | | |
| - K57.2 (Diverticular disease of large intestine with perforation and abscess) - K57.3 (Diverticular disease of large intestine without perforation or abscess) | Days 1–28 | Cases diagnosed with colonic diverticulitis within 365 days prior to the cohort entry date.   - K57.* (Diverticular disease of intestine) |
|  | Inpatient; Emergency department |  |

Supplemental Table S4. Operational definitions of adverse events of interest and negative control events for near-real time sequential monitoring

Abbreviation: KCD-8, Korean Standard Classification of Diseases-8th Revision.

^a^ The cohort entry date was defined as January 1 of each year during the study period or the first date meeting the clean period requirement for background incidence rate analysis, and as the vaccination date for sequential analysis.

[1] Moll K, Lufkin B, Fingar KR, Ke Zhou C, Tworkoski E, Shi C, et al. Background rates of adverse events of special interest for COVID-19 vaccine safety monitoring in the United States, 2019-2020. Vaccine. 2023;41(2):333-353. doi: 10.1016/j.vaccine.2022.11.003.

[2] Klein NP, Lewis N, Goddard K, Fireman B, Zerbo O, Hanson KE, et al. Surveillance for Adverse Events After COVID-19 mRNA Vaccination. JAMA. 2021;326(14):1390-1399. doi: 10.1001/jama.2021.15072.

[3] Kimm H, Yun JE, Lee SH, Jang Y, Jee SH. Validity of the diagnosis of acute myocardial infarction in korean national medical health insurance claims data: the korean heart study (1). Korean Circ J. 2012;42(1):10-5. doi: 10.4070/kcj.2012.42.1.10.

[4] Phillips A, Jiang Y, Walsh D, Andrews N, Artama M, Clothier H, et al. Background rates of adverse events of special interest for COVID-19 vaccines: A multinational Global Vaccine Data Network (GVDN) analysis. Vaccine. 2023;41(42):6227-6238. doi: 10.1016/j.vaccine.2023.08.079.

[5] Choe YJ, Lee H, Kim JH, Choi WS, Shin JY. Anaphylaxis following vaccination among children in Asia: A large-linked database study. Allergy. 2021;76(4):1246-1249. doi: 10.1111/all.14562.

[6] Li D, Baxter NN, McLeod RS, Moineddin R, Wilton AS, Nathens AB. Evolving practice patterns in the management of acute colonic diverticulitis: a population-based analysis. Dis Colon Rectum. 2014;57(12):1397-405. doi: 10.1097/DCR.0000000000000224.

Supplemental Table S5. Pre-specified relative risks under the null hypothesis for each adverse event of interest for sequential monitoring

| **Adverse events of special interest** | **Relative risk** |
| --- | --- |
| Acute myocardial infarction | 1.25 |
| Myocarditis | 1.5 |
| Anaphylaxis | 1.5 |
| Colonic diverticulitis | 1.5 |

Supplemental Table S6. Data delay distribution used for calculating follow-up periods for adverse events of special interest

| **Adverse events of special interest** | **Data accrual rate** | | | |
| --- | --- | --- | --- | --- |
|  | **Diagnosis month** | **1 month after diagnosis** | **2 months after diagnosis** | **3 months after diagnosis** |
| Acute myocardial infarction | 0% | 2.8% | 79.4% | 96.1% |
| Myocarditis | 0% | 4.7% | 79.5% | 95.9% |
| Anaphylaxis | 0% | 7.1% | 78.1% | 98.6% |
| Colonic diverticulitis | 0% | 4.4% | 78.9% | 96.5% |

Supplemental Table S7. Results of sequential testing of the risk of acute myocardial infarction

| Vaccine platform and dose | | At data cutoff point | | | | At statistical signal detection point | | | | |
| --- | --- | --- | --- | --- | --- | --- | --- | --- | --- | --- |
|  |  | Person-days | Events | RR | LLR^a^ | Statistical signal | Date | Events | RR | LLR |
| **12–17 years** | | | | | | |  |  |  |  |
| mRNA | Dose 1 | 63,541,922 | 13 | 1.53 | 0.24 | No | - | - | - | - |
|  | Dose 2 | 60,245,668 | 9 | 1.12 | 0 | No | - | - | - | - |
|  | Dose 3 | 7,880,927 | 1 | 0.95 | 0 | No | - | - | - | - |
| Recombinant protein-based | Dose 1 | 224 | 0 | 0 | 0 | No | - | - | - | - |
|  | Dose 2 | 28 | 0 | 0 | 0 | No | - | - | - | - |
|  | Dose 3 | 30 | 0 | 0 | 0 | No | - | - | - | - |
| mRNA (bivalent) | | 902,192 | 0 | 0 | 0 | No | - | - | - | - |
| **18–39 years** | | | | | | |  |  |  |  |
| mRNA | Dose 1 | 317,925,229 | 98 | 0.87 | 0 | No | - | - | - | - |
|  | Dose 2 | 327,214,948 | 104 | 0.89 | 0 | No | - | - | - | - |
|  | Dose 3 | 215,673,540 | 54 | 0.70 | 0 | No | - | - | - | - |
| Non-replicating viral vector | Dose 1 | 43,203,797 | 13 | 0.84 | 0 | No | - | - | - | - |
|  | Dose 2 | 7,546,332 | 1 | 0.37 | 0 | No | - | - | - | - |
| Recombinant protein-based | Dose 1 | 1,329,832 | 2 | 4.26 | 1.04 | No | - | - | - | - |
|  | Dose 2 | 980,578 | 0 | 0 | 0 | No | - | - | - | - |
|  | Dose 3 | 605,688 | 1 | 4.55 | 0.57 | No | - | - | - | - |
| mRNA (bivalent) | | 18,459,167 | 9 | 1.37 | 0.04 | No | - | - | - | - |
| **40–64 years** | | | | | | |  |  |  |  |
| mRNA | Dose 1 | 380,160,417 | 650 | 0.65 | 0 | No | - | - | - | - |
|  | Dose 2 | 411,212,481 | 719 | 0.66 | 0 | No | - | - | - | - |
|  | Dose 3 | 441,870,405 | 881 | 0.76 | 0 | No | - | - | - | - |
| Non-replicating viral vector | Dose 1 | 162,362,381 | 480 | 1.12 | 0 | No | - | - | - | - |
|  | Dose 2 | 116,882,602 | 327 | 1.06 | 0 | No | - | - | - | - |
| Recombinant protein-based | Dose 1 | 1,425,914 | 3 | 0.80 | 0 | No | - | - | - | - |
|  | Dose 2 | 1,190,953 | 0 | 0 | 0 | No | - | - | - | - |
|  | Dose 3 | 1,135,323 | 1 | 0.33 | 0 | No | - | - | - | - |
| mRNA (bivalent) | | 56,588,779 | 159 | 1.06 | 0 | No | - | - | - | - |
| **≥65 years** | | | | | | |  |  |  |  |
| mRNA | Dose 1 | 98,829,442 | 710 | 0.68 | 0 | No | - | - | - | - |
|  | Dose 2 | 101,120,078 | 748 | 0.7 | 0 | No | - | - | - | - |
|  | Dose 3 | 220,226,137 | 1,329 | 0.57 | 0 | No | - | - | - | - |
| Non-replicating viral vector | Dose 1 | 133,355,503 | 735 | 0.52 | 0 | No | - | - | - | - |
|  | Dose 2 | 128,274,804 | 613 | 0.45 | 0 | No | - | - | - | - |
| Recombinant protein-based | Dose 1 | 557,734 | 5 | 0.85 | 0 | No | - | - | - | - |
|  | Dose 2 | 488,684 | 1 | 0.39 | 0 | No | - | - | - | - |
|  | Dose 3 | 297,271 | 0 | 0 | 0 | No | - | - | - | - |
| mRNA (bivalent) | | 99,718,650 | 581 | 0.55 | 0 | No | - | - | - | - |

Abbreviation: RR, relative risk; LLR, log-likelihood ratio

^a^ The log-likelihood ratio is artificially set to 0 when the relative risk is less than 1.

Supplemental Table S8. Results of sequential testing of the risk of colonic diverticulitis

| Vaccine platform and dose | | At data cutoff point | | | | At statistical signal detection point | | | | |
| --- | --- | --- | --- | --- | --- | --- | --- | --- | --- | --- |
|  |  | Person-days | Events | RR | LLR^a^ | Statistical signal | Date | Events | RR | LLR |
| **12–17 years** | | | | | | |  |  |  |  |
| mRNA | Dose 1 | 63,759,308 | 13 | 1.15 | 0 | No | - | - | - | - |
|  | Dose 2 | 60,246,262 | 14 | 1.31 | 0 | No | - | - | - | - |
|  | Dose 3 | 7,884,098 | 2 | 1.43 | 0 | No | - | - | - | - |
| Recombinant protein-based | Dose 1 | 225 | 0 | 0 | 0 | No | - | - | - | - |
|  | Dose 2 | 28 | 0 | 0 | 0 | No | - | - | - | - |
|  | Dose 3 | 31 | 0 | 0 | 0 | No | - | - | - | - |
| mRNA (bivalent) | | 902,415 | 1 | 6.25 | 0.67 | No | - | - | - | - |
| **18–39 years** | | | | | | |  |  |  |  |
| mRNA | Dose 1 | 317,924,601 | 225 | 0.82 | 0 | No | - | - | - | - |
|  | Dose 2 | 327,214,542 | 239 | 0.85 | 0 | No | - | - | - | - |
|  | Dose 3 | 215,679,720 | 142 | 0.77 | 0 | No | - | - | - | - |
| Non-replicating viral vector | Dose 1 | 43,203,370 | 60 | 1.62 | 0.17 | No | - | - | - | - |
|  | Dose 2 | 7,546,276 | 6 | 0.93 | 0 | No | - | - | - | - |
| Recombinant protein-based | Dose 1 | 1,330,317 | 2 | 1.75 | 0.02 | No | - | - | - | - |
|  | Dose 2 | 981,020 | 1 | 1.19 | 0 | No | - | - | - | - |
|  | Dose 3 | 606,654 | 1 | 1.92 | 0.03 | No | - | - | - | - |
| mRNA (bivalent) | | 18,465,876 | 9 | 0.57 | 0 | No | - | - | - | - |
| **40–64 years** | | | | | | |  |  |  |  |
| mRNA | Dose 1 | 380,163,941 | 479 | 0.90 | 0 | No | - | - | - | - |
|  | Dose 2 | 411,216,380 | 483 | 0.84 | 0 | No | - | - | - | - |
|  | Dose 3 | 441,887,223 | 481 | 0.77 | 0 | No | - | - | - | - |
| Non-replicating viral vector | Dose 1 | 162,368,339 | 269 | 1.18 | 0 | No | - | - | - | - |
|  | Dose 2 | 116,889,831 | 161 | 0.98 | 0 | No | - | - | - | - |
| Recombinant protein-based | Dose 1 | 1,426,268 | 5 | 2.50 | 0.55 | No | - | - | - | - |
|  | Dose 2 | 1,191,256 | 0 | 0 | 0 | No | - | - | - | - |
|  | Dose 3 | 1,136,982 | 2 | 1.25 | 0 | No | - | - | - | - |
| mRNA (bivalent) | | 56,599,685 | 56 | 0.70 | 0 | No | - | - | - | - |
| **≥65 years** | | | | | | |  |  |  |  |
| mRNA | Dose 1 | 98,844,243 | 183 | 1.32 | 0 | No | - | - | - | - |
|  | Dose 2 | 101,136,247 | 180 | 1.27 | 0 | No | - | - | - | - |
|  | Dose 3 | 220,273,978 | 282 | 0.91 | 0 | No | - | - | - | - |
| Non-replicating viral vector | Dose 1 | 133,396,278 | 197 | 1.05 | 0 | No | - | - | - | - |
|  | Dose 2 | 128,310,619 | 155 | 0.86 | 0 | No | - | - | - | - |
| Recombinant protein-based | Dose 1 | 558,451 | 1 | 1.28 | 0 | No | - | - | - | - |
|  | Dose 2 | 488,935 | 0 | 0 | 0 | No | - | - | - | - |
|  | Dose 3 | 298,063 | 0 | 0 | 0 | No | - | - | - | - |
| mRNA (bivalent) | | 99,749,237 | 126 | 0.90 | 0 | No | - | - | - | - |

Abbreviation: RR, relative risk; LLR, log-likelihood ratio

^a^ The log-likelihood ratio is artificially set to 0 when the relative risk is less than 1.

Supplemental Table S9. Results of sequential testing of the risk of myocarditis

| Vaccine platform and dose | | At data cutoff point | | | | At statistical signal detection point | | | | |
| --- | --- | --- | --- | --- | --- | --- | --- | --- | --- | --- |
|  |  | Person-days | Events | RR | LLR^a^ | Statistical signal | Date | Events | RR | LLR |
| **12–17 years** | | | | | | |  |  |  |  |
| mRNA | Dose 1 | 69,097,626 | 54 | 3.38 | 13.83 | Yes | Jan 31, 2022 | 17 | 3.79 | 5.48 |
|  | Dose 2 | 70,515,241 | 54 | 3.31 | 13.22 | Yes | Jan 31, 2022 | 12 | 6.70 | 8.65 |
|  | Dose 3 | 7,720,439 | 12 | 6.70 | 8.65 | Yes | Jun 30, 2022 | 8 | 11.27 | 9.20 |
| Recombinant protein-based | Dose 1 | 244 | 0 | 0 | 0 | No | - | - | - | - |
|  | Dose 2 | 11 | 0 | 0 | 0 | No | - | - | - | - |
|  | Dose 3 | 44 | 0 | 0 | 0 | No | - | - | - | - |
| mRNA (bivalent) | | 916,588 | 1 | 4.76 | 0.47 | No | - | - | - | - |
| **18–39 years** | | | | | | |  |  |  |  |
| mRNA | Dose 1 | 349,949,622 | 146 | 7.89 | 124.16 | Yes | Sep 30, 2021 | 11 | 6.92 | 8.20 |
|  | Dose 2 | 375,637,912 | 115 | 5.79 | 70.13 | Yes | Sep 30, 2021 | 7 | 9.33 | 6.92 |
|  | Dose 3 | 233,868,158 | 32 | 2.59 | 3.99 | Yes | Feb 28, 2022 | 11 | 5.21 | 5.87 |
| Non-replicating viral vector | Dose 1 | 57,850,651 | 2 | 0.65 | 0 | No | - | - | - | - |
|  | Dose 2 | 6,349,606 | 1 | 2.94 | 0.18 | No | - | - | - | - |
| Recombinant protein-based | Dose 1 | 1,509,741 | 1 | 12.5 | 1.24 | No | - | - | - | - |
|  | Dose 2 | 1,146,001 | 0 | 0 | 0 | No | - | - | - | - |
|  | Dose 3 | 623,968 | 0 | 0 | 0 | No | - | - | - | - |
| mRNA (bivalent) | | 20,475,036 | 1 | 0.93 | 0 | No | - | - | - | - |
| **40–64 years** | | | | | | |  |  |  |  |
| mRNA | Dose 1 | 408,331,078 | 61 | 2.41 | 5.92 | Yes | Dec 31, 2021 | 47 | 2.10 | 2.40 |
|  | Dose 2 | 473,596,493 | 53 | 1.81 | 0 | No | - | - | - | - |
|  | Dose 3 | 482,669,632 | 30 | 1.00 | 0 | No | - | - | - | - |
| Non-replicating viral vector | Dose 1 | 215,602,959 | 9 | 0.67 | 0 | No | - | - | - | - |
|  | Dose 2 | 95,589,063 | 7 | 1.18 | 0 | No | - | - | - | - |
| Recombinant protein-based | Dose 1 | 1,676,909 | 3 | 30.00 | 6.14 | Yes | Jun 30, 2022 | 2 | 22.22 | 3.53 |
|  | Dose 2 | 1,386,915 | 3 | 33.33 | 6.44 | Yes | Jun 30, 2022 | 2 | 40.00 | 6.64 |
|  | Dose 3 | 1,184,126 | 0 | 0 | 0 | No | - | - | - | - |
| mRNA (bivalent) | | 61,864,894 | 0 | 0 | 0 | No | - | - | - | - |
| **≥65 years** | | | | | | |  |  |  |  |
| mRNA | Dose 1 | 101,019,575 | 10 | 0.73 | 0 | No | - | - | - | - |
|  | Dose 2 | 108,124,399 | 12 | 0.82 | 0 | No | - | - | - | - |
|  | Dose 3 | 269,120,179 | 42 | 1.15 | 0 | No | - | - | - | - |
| Non-replicating viral vector | Dose 1 | 153,387,233 | 4 | 0.19 | 0 | No | - | - | - | - |
|  | Dose 2 | 148,439,986 | 9 | 0.45 | 0 | No | - | - | - | - |
| Recombinant protein-based | Dose 1 | 644,669 | 0 | 0 | 0 | No | - | - | - | - |
|  | Dose 2 | 553,253 | 0 | 0 | 0 | No | - | - | - | - |
|  | Dose 3 | 310,418 | 0 | 0 | 0 | No | - | - | - | - |
| mRNA (bivalent) | | 109,511,038 | 23 | 1.54 | 0.01 | No | - | - | - | - |

Abbreviation: RR, relative risk; LLR, log-likelihood ratio

^a^ The log-likelihood ratio is artificially set to 0 when the relative risk is less than 1.

Supplemental Table S10. Results of sequential testing of the risk of anaphylaxis

| Vaccine platform and dose | | At data cutoff point | | | | At statistical signal detection point | | | | |  |
| --- | --- | --- | --- | --- | --- | --- | --- | --- | --- | --- | --- |
|  |  | Person-days | Events | RR | LLR^a^ | Statistical signal | Date | Events | RR | LLR |  |
| **12–17 years** | | | | | | |  |  |  |  |  |
| mRNA | Dose 1 | 2,277,988 | 4 | 2.72 | 0.59 | No | - | - | - | - |  |
|  | Dose 2 | 2,152,688 | 3 | 2.16 | 0.18 | No | - | - | - | - |  |
|  | Dose 3 | 286,179 | 0 | 0 | 0 | No | - | - | - | - |  |
| Recombinant protein-based | Dose 1 | 7 | 0 | 0 | 0 | No | - | - | - | - |  |
|  | Dose 2 | 1 | 0 | 0 | 0 | No | - | - | - | - |  |
|  | Dose 3 | 2 | 0 | 0 | 0 | No | - | - | - | - |  |
| mRNA (bivalent) | | 33,544 | 0 | 0 | 0 | No | - | - | - | - |  |
| **18–39 years** | | | | | | |  |  |  |  |  |
| mRNA | Dose 1 | 11,355,193 | 133 | 17.30 | 203.72 | Yes | Jun 30, 2021 | 2 | 66.67 | 5.63 |  |
|  | Dose 2 | 11,687,130 | 44 | 5.56 | 25.49 | Yes | Aug 31, 2021 | 3 | 25.00 | 5.62 |  |
|  | Dose 3 | 7,713,760 | 13 | 2.49 | 1.42 | Yes | Jan 31, 2022 | 4 | 9.30 | 3.94 |  |
| Non-replicating viral vector | Dose 1 | 1,543,045 | 43 | 40.95 | 100.77 | Yes | Apr 30, 2021 | 9 | 900.00 | 48.59 |  |
|  | Dose 2 | 269,520 | 4 | 27.32 | 9.79 | Yes | Jul 31, 2021 | 2 | 20.00 | 3.33 |  |
| Recombinant protein-based | Dose 1 | 48,224 | 0 | 0 | 0 | No | - | - | - | - |  |
|  | Dose 2 | 35,704 | 0 | 0 | 0 | No | - | - | - | - |  |
|  | Dose 3 | 43,085 | 0 | 0 | 0 | No | - | - | - | - |  |
| mRNA (bivalent) | | 684,740 | 0 | 0 | 0 | No | - | - | - | - |  |
| **40–64 years** | | | | | | |  |  |  |  |  |
| mRNA | Dose 1 | 13,578,335 | 105 | 6.14 | 68.59 | Yes | Jun 30, 2021 | 3 | 42.86 | 7.16 | |
|  | Dose 2 | 14,686,963 | 48 | 2.59 | 6.04 | Yes | Jul 31, 2021 | 2 | 28.57 | 4.00 | |
|  | Dose 3 | 15,794,614 | 12 | 0.60 | 0 | No | - | - | - | - | |
| Non-replicating viral vector | Dose 1 | 5,799,204 | 64 | 8.76 | 59.87 | Yes | Apr 30, 2021 | 3 | 42.86 | 7.16 | |
|  | Dose 2 | 4,173,320 | 11 | 2.09 | 0.55 | Yes | Jul 31, 2021 | 2 | 28.57 | 4.00 | |
| Recombinant protein-based | Dose 1 | 51,461 | 0 | 0 | 0 | No | - | - | - | - |  |
|  | Dose 2 | 43,085 | 0 | 0 | 0 | No | - | - | - | - |  |
|  | Dose 3 | 43,212 | 0 | 0 | 0 | No | - | - | - | - |  |
| mRNA (bivalent) | | 2,048,577 | 2 | 0.78 | 0 | No | - | - | - | - |  |
| **≥65 years** | | | | | | |  |  |  |  |  |
| mRNA | Dose 1 | 3,147,253 | 23 | 5.35 | 12.69 | Yes | Jul 31, 2021 | 14 | 5.69 | 8.36 |  |
|  | Dose 2 | 3,613,725 | 15 | 3.41 | 3.91 | Yes | Aug 31, 2021 | 10 | 3.25 | 2.34 |  |
|  | Dose 3 | 7,869,763 | 5 | 0.52 | 0 | No | - | - | - | - |  |
| Non-replicating viral vector | Dose 1 | 4,759,853 | 16 | 2.76 | 2.45 | Yes | Jul 31, 2021 | 7 | 3.65 | 2.10 |  |
|  | Dose 2 | 4,578,155 | 9 | 1.61 | 0.02 | No | - | - | - | - |  |
| Recombinant protein-based | Dose 1 | 20,356 | 0 | 0 | 0 | No | - | - | - | - |  |
|  | Dose 2 | 17,808 | 0 | 0 | 0 | No | - | - | - | - |  |
|  | Dose 3 | 12,019 | 0 | 0 | 0 | No | - | - | - | - |  |
| mRNA (bivalent) | | 3,586,826 | 2 | 0.46 | 0 | No | - | - | - | - |  |

Abbreviation: RR, relative risk; LLR, log-likelihood ratio

^a^ The log-likelihood ratio is artificially set to 0 when the relative risk is less than 1.

Supplemental Table S11. Sensitive analysis results of sequential testing of the risk of myocarditis

| Vaccine platform and dose | | At data cutoff point | | | | At statistical signal detection point | | | | |
| --- | --- | --- | --- | --- | --- | --- | --- | --- | --- | --- |
|  |  | Person-days | Events | RR | LLR^a^ | Statistical signal | Date | Events | RR | LLR |
| **12–17 years** | | | | | | |  |  |  |  |
| mRNA | Dose 1 | 69,097,626 | 54 | 3.50 | 14.93 | Yes | Jan 31, 2022 | 17 | 3.82 | 5.57 |
|  | Dose 2 | 70,515,241 | 54 | 3.34 | 13.47 | Yes | Jan 31, 2022 | 12 | 6.56 | 8.45 |
|  | Dose 3 | 7,720,439 | 12 | 7.50 | 9.71 | Yes | Jun 30, 2022 | 8 | 14.04 | 10.74 |
| **18–39 years** | | | | | | |  |  |  |  |
| mRNA | Dose 1 | 349,949,622 | 146 | 8.14 | 127.88 | Yes | Sep 30, 2021 | 11 | 7.64 | 9.07 |
|  | Dose 2 | 375,637,912 | 115 | 5.66 | 68.18 | Yes | Sep 30, 2021 | 7 | 10.94 | 7.87 |
|  | Dose 3 | 233,868,158 | 32 | 2.42 | 3.12 | Yes | Feb 28, 2022 | 11 | 4.54 | 4.76 |
| **40–64 years** | | | | | | |  |  |  |  |
| mRNA | Dose 1 | 408,331,078 | 61 | 2.34 | 5.23 | Yes | Dec 31, 2021 | 47 | 2.26 | 3.48 |
| Recombinant protein-based | Dose 1 | 1,676,909 | 3 | 25.00 | 5.62 | Yes | Jun 30, 2022 | 2 | 25.00 | 3.75 |
|  | Dose 2 | 1,386,915 | 3 | 33.33 | 6.44 | Yes | Jun 30, 2022 | 2 | 40.00 | 4.64 |

Abbreviation: RR, relative risk; LLR, log-likelihood ratio

^a^ The log-likelihood ratio is artificially set to 0 when the relative risk is less than 1.

Supplemental Table S12. Sensitive analysis results of sequential testing of the risk of anaphylaxis

| Vaccine platform and dose | | At data cutoff point | | | | At statistical signal detection point | | | | |  |
| --- | --- | --- | --- | --- | --- | --- | --- | --- | --- | --- | --- |
|  |  | Person-days | Events | RR | LLR^a^ | Statistical signal | Date | Events | RR | LLR |  |
| **18–39 years** | | | | | | |  |  |  |  |  |
| mRNA | Dose 1 | 11,355,193 | 133 | 18.65 | 212.93 | Yes | Jun 30, 2021 | 2 | 66.67 | 5.63 |  |
|  | Dose 2 | 11,687,130 | 44 | 5.96 | 27.79 | Yes | Aug 31, 2021 | 3 | 27.27 | 5.87 |  |
|  | Dose 3 | 7,713,760 | 13 | 2.79 | 2.06 | Yes | Jan 31, 2022 | 4 | 12.50 | 4.96 |  |
| Non-replicating viral vector | Dose 1 | 1,543,045 | 43 | 44.33 | 104.06 | Yes | Apr 30, 2021 | 9 | 900.00 | 48.59 |  |
|  | Dose 2 | 269,520 | 5 | 27.78 | 9.86 | Yes | Jul 31, 2021 | 2 | 22.22 | 3.53 |  |
| **40–64 years** | | | | | | |  |  |  |  |  |
| mRNA | Dose 1 | 13,578,335 | 105 | 5.83 | 64.56 | Yes | Jun 30, 2021 | 3 | 50.00 | 7.61 | |
|  | Dose 2 | 14,686,963 | 48 | 2.27 | 3.41 | Yes | Jul 31, 2021 | 2 | 25.00 | 3.75 | |
| Non-replicating viral vector | Dose 1 | 5,799,204 | 64 | 8.32 | 57.2 | Yes | Apr 30, 2021 | 4 | 100.00 | 12.86 | |
|  | Dose 2 | 4,174,877 | 11 | 1.96 | 0.36 | No | - | - | - | - | |
| **≥65 years** | | | | | | |  |  |  |  |  |
| mRNA | Dose 1 | 3,531,996 | 23 | 5.07 | 11.8 | Yes | Jul 31, 2021 | 14 | 5.60 | 8.19 |  |
|  | Dose 2 | 3,613,725 | 15 | 3.18 | 3.36 | Yes | Sep 30, 2021 | 15 | 3.29 | 3.62 |  |
| Non-replicating viral vector | Dose 1 | 4,759,853 | 16 | 2.48 | 1.71 | No | - | - | - | - |  |

Abbreviation: RR, relative risk; LLR, log-likelihood ratio

^a^ The log-likelihood ratio is artificially set to 0 when the relative risk is less than 1.


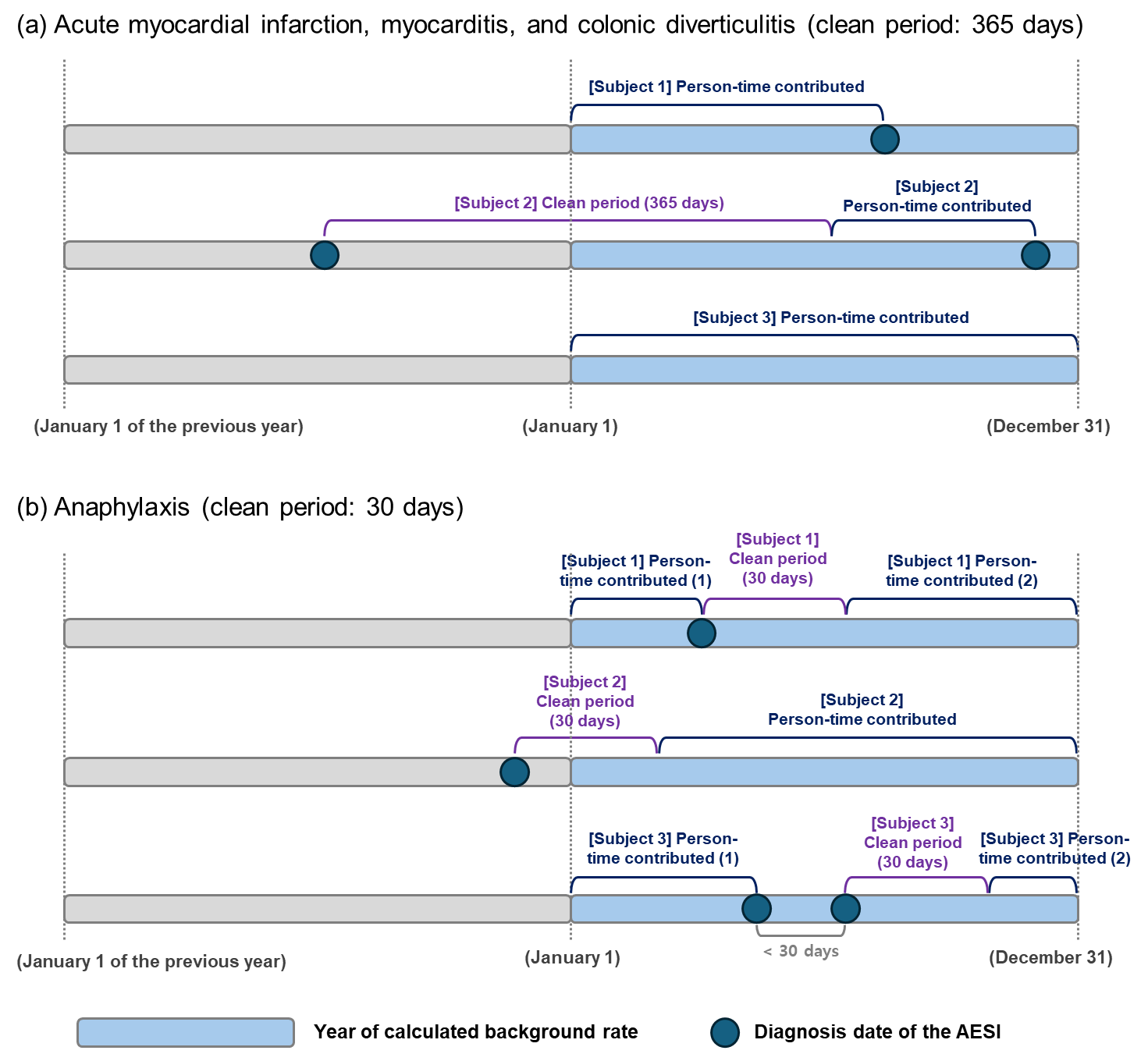


Supplemental Figure S1. Diagram for calculating background rates.

* The clean period was defined as the time window prior to study entry during which the AESI was required to be absent, serving as a prerequisite for inclusion in the cohort. The specific duration varied depending on each AESI and negative control event.


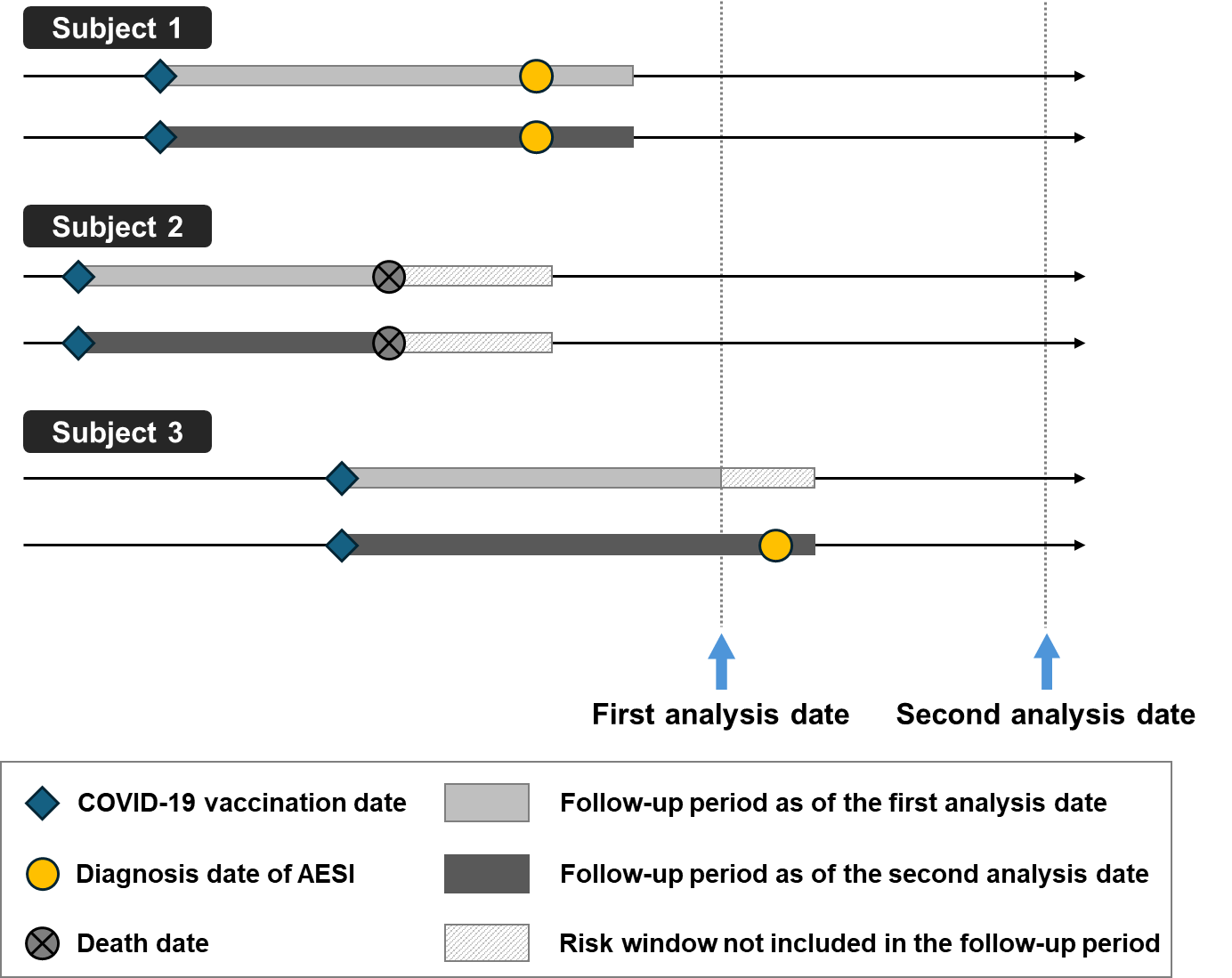


Supplemental Figure S2. Diagram of the follow-up period in sequential analysis.

* Subject 1: The individual had completed the risk period for each AESI as of the first analysis date. They contributed the entire risk period to the follow-up period for both the first and subsequent analyses, and outcomes were counted if they occurred.

* Subject 2: The risk period was censored due to death. This individual contributed to the observation period only up to the censoring point for both the first and subsequent analyses.

* Subject 3: At the first analysis date, the risk window had not yet ended and was censored on that date. The individual contributed to the follow-up period up to the censoring date at the first analysis, while the entire pre-defined risk window was included in the follow-up period for subsequent analyses. Outcomes were not counted at the first analysis but were counted in subsequent analyses.


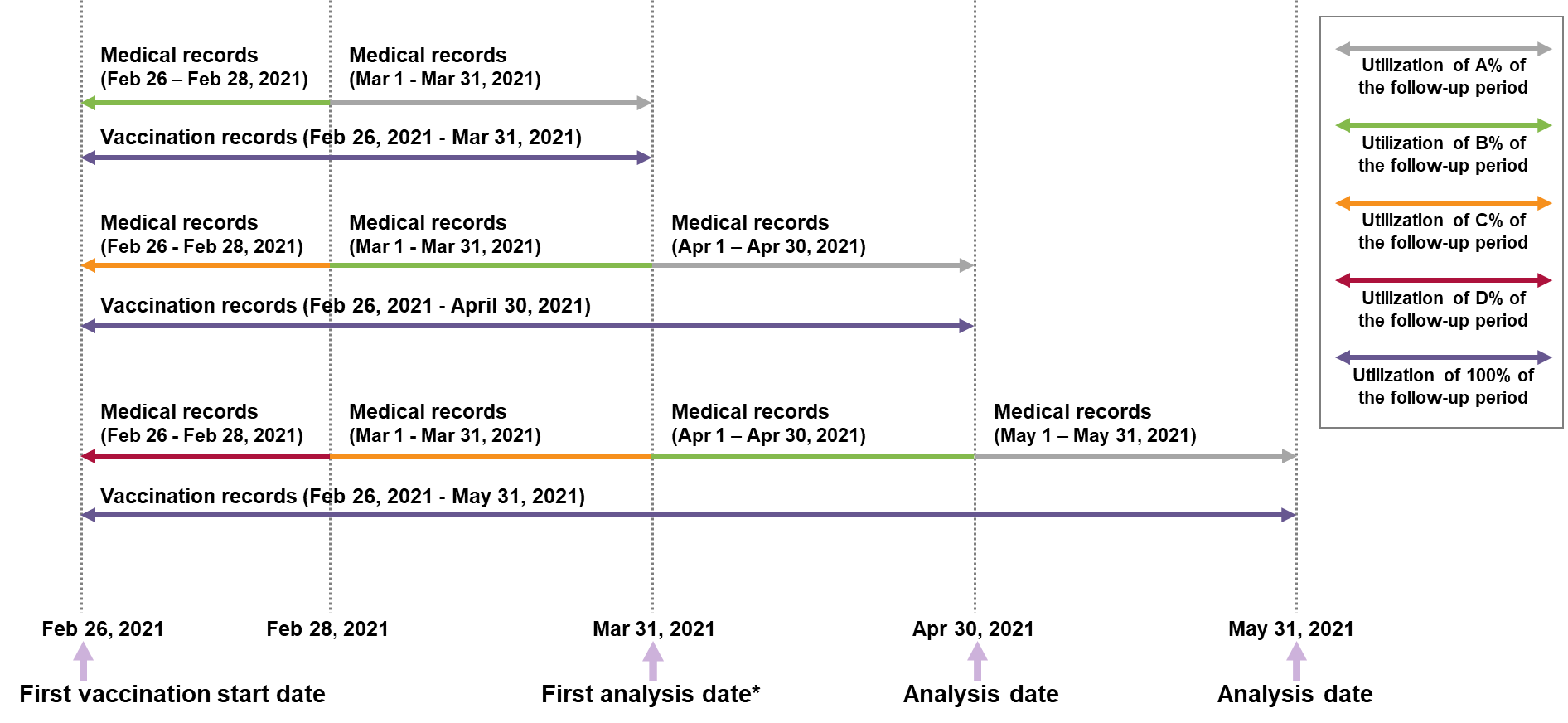


Supplemental Figure S3. Diagram for calculating the follow-up period accounting for data delays in sequential analysis.

* This outlines the proportion of medical records for a given month that were accrued into the database by specific time points: A% by the end of the same month, B% by the end of the following month, C% by the end of the second month, and D% by the end of the third month.

* The follow-up period was fully utilized after the point when more than 95% of claims for specific outcomes in a specific month of care were fully accrued.

* The analysis begins when at least one case is observed at the time of analysis. If the cumulative number of cases is less than one on the scheduled analysis date, the analysis will be postponed and initiated at the next scheduled time point when at least one case has been identified.


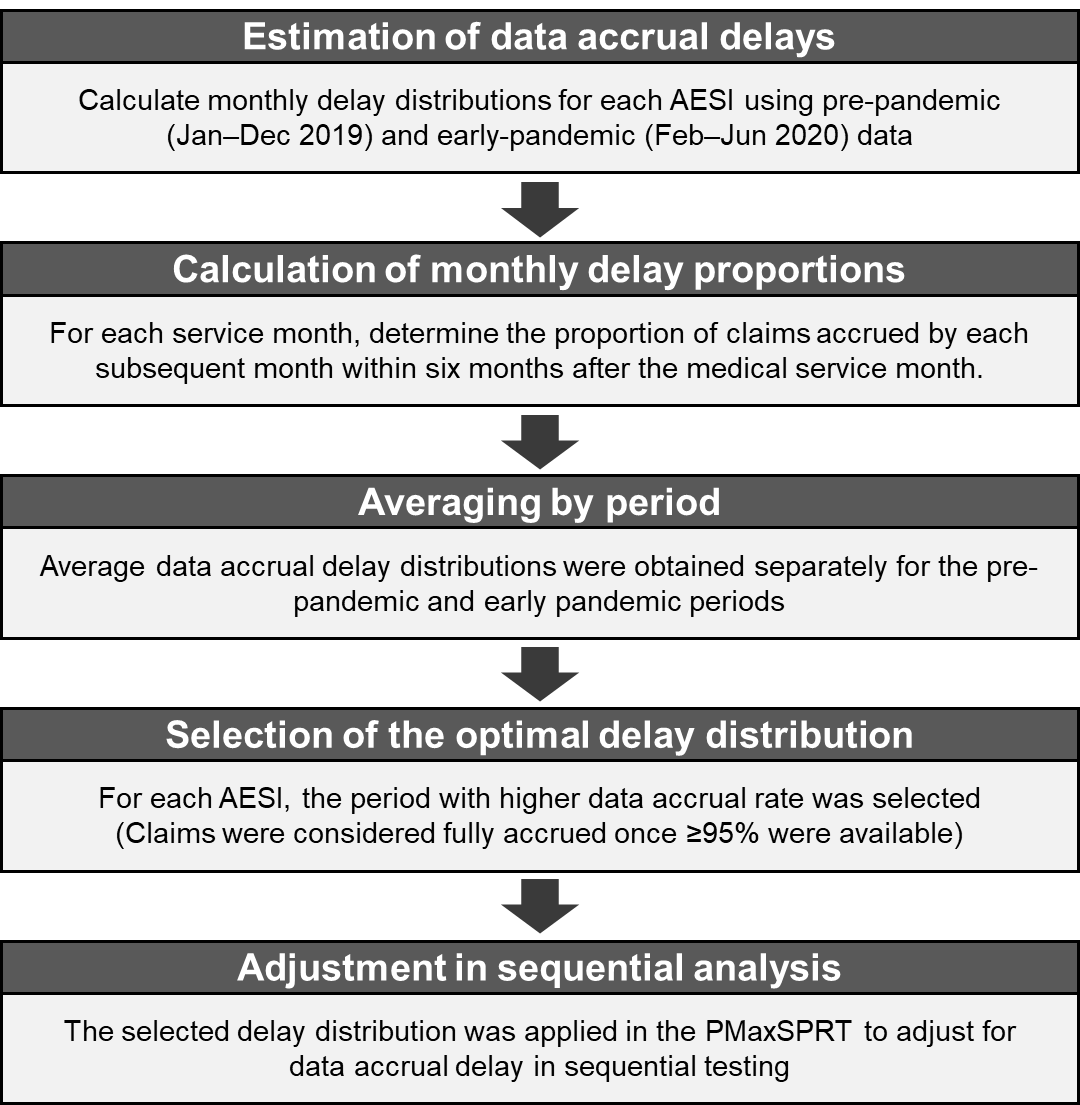


Supplemental Figure S4. Process for adjusting data accrual delays in claims data.


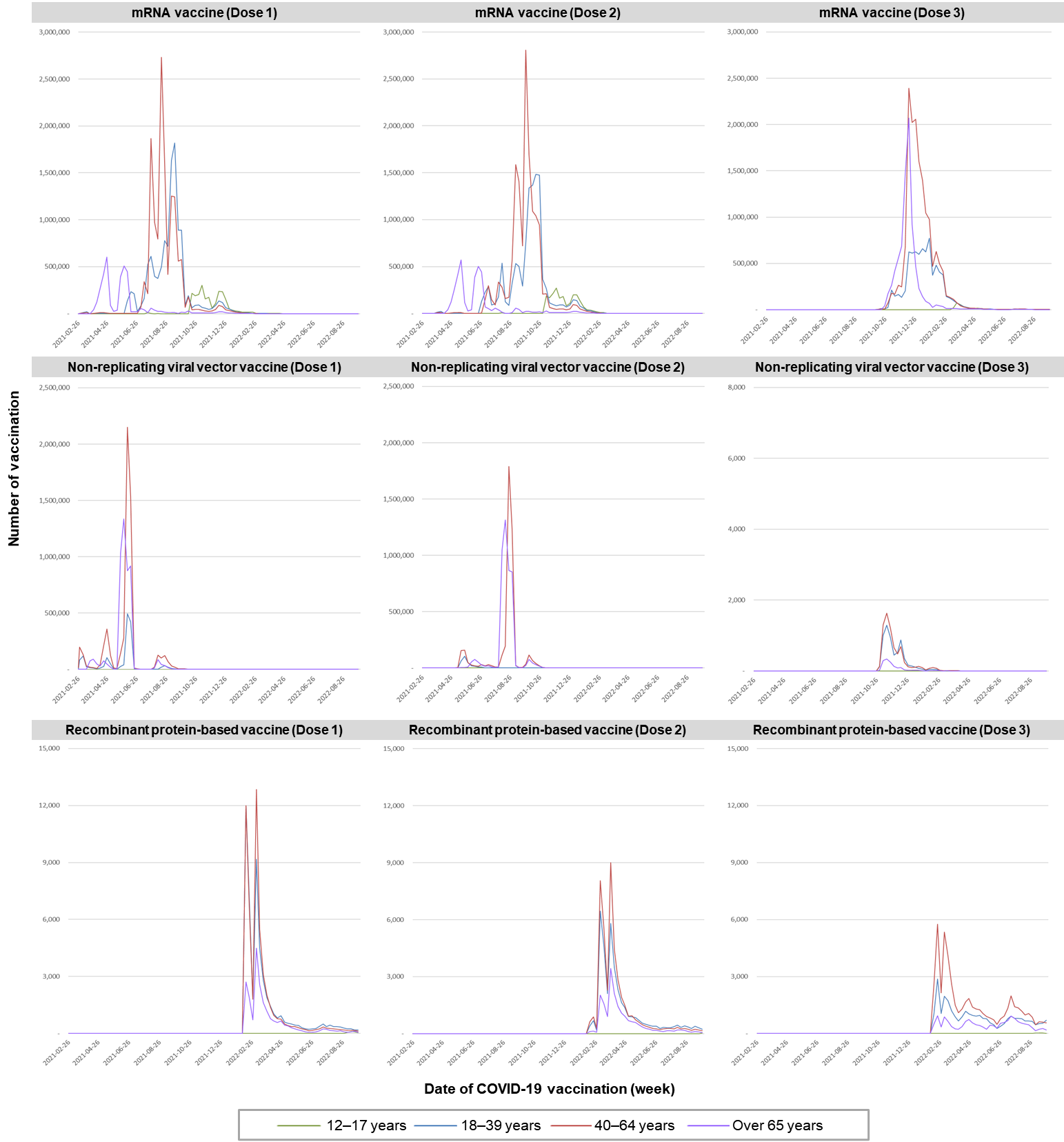


Supplemental Figure S5. Weekly COVID-19 monovalent vaccine administration by age group and vaccine type.


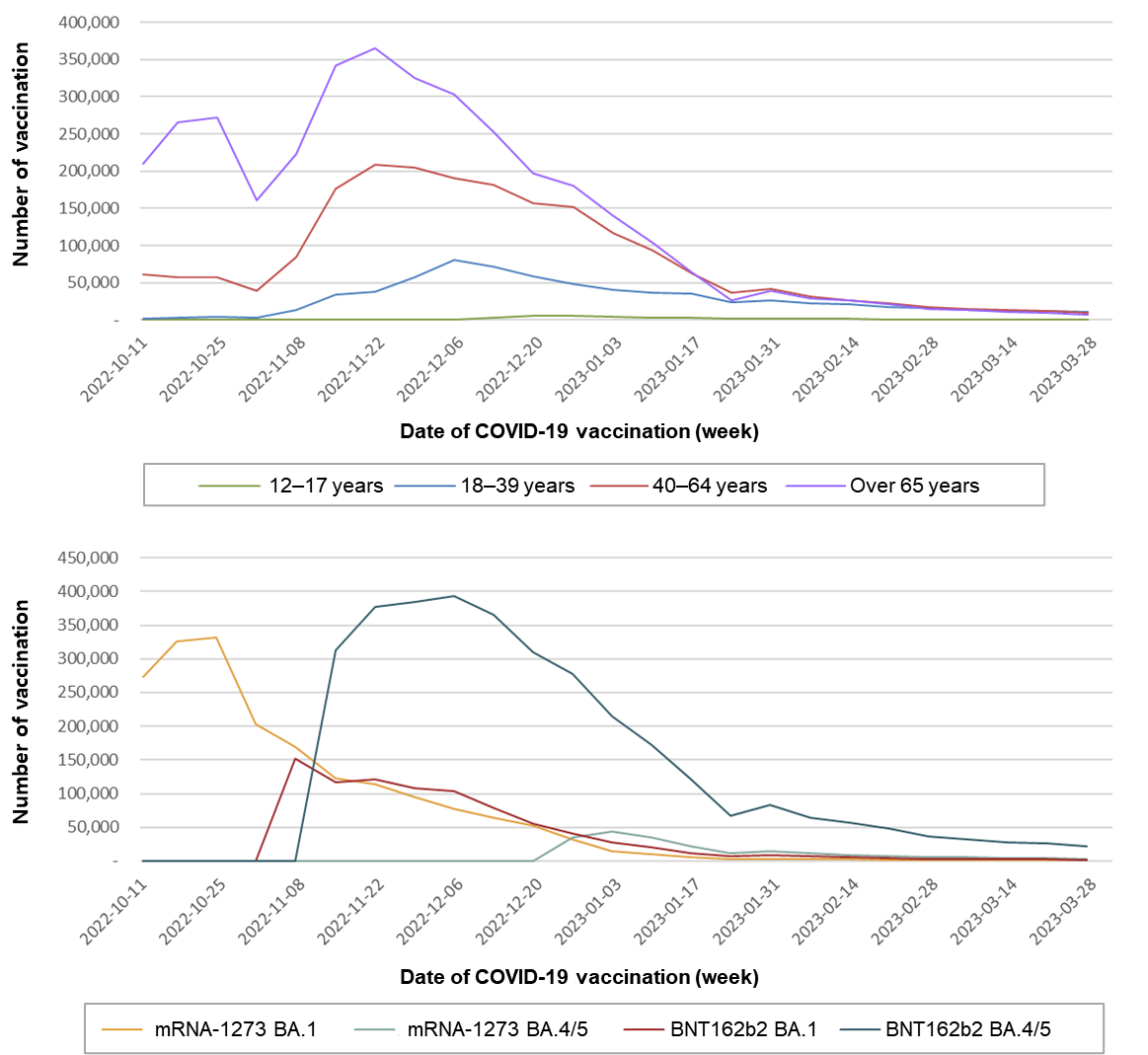


Supplemental Figure S6. Weekly COVID-19 bivalent vaccine administration by age group and vaccine type.


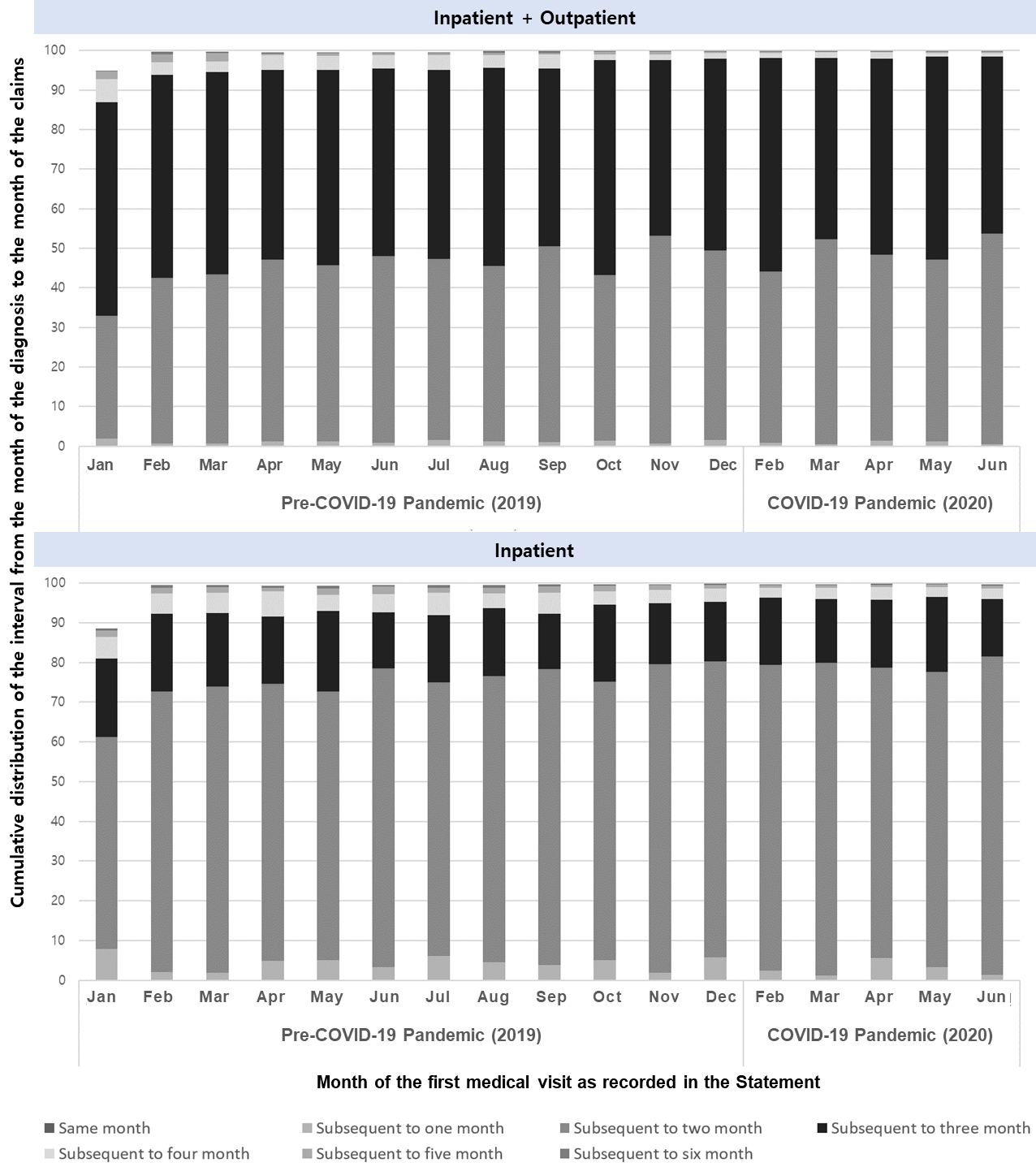


Supplemental Figure S7. Distribution of the interval between diagnosis month of acute myocardial infarction and data accrual month.


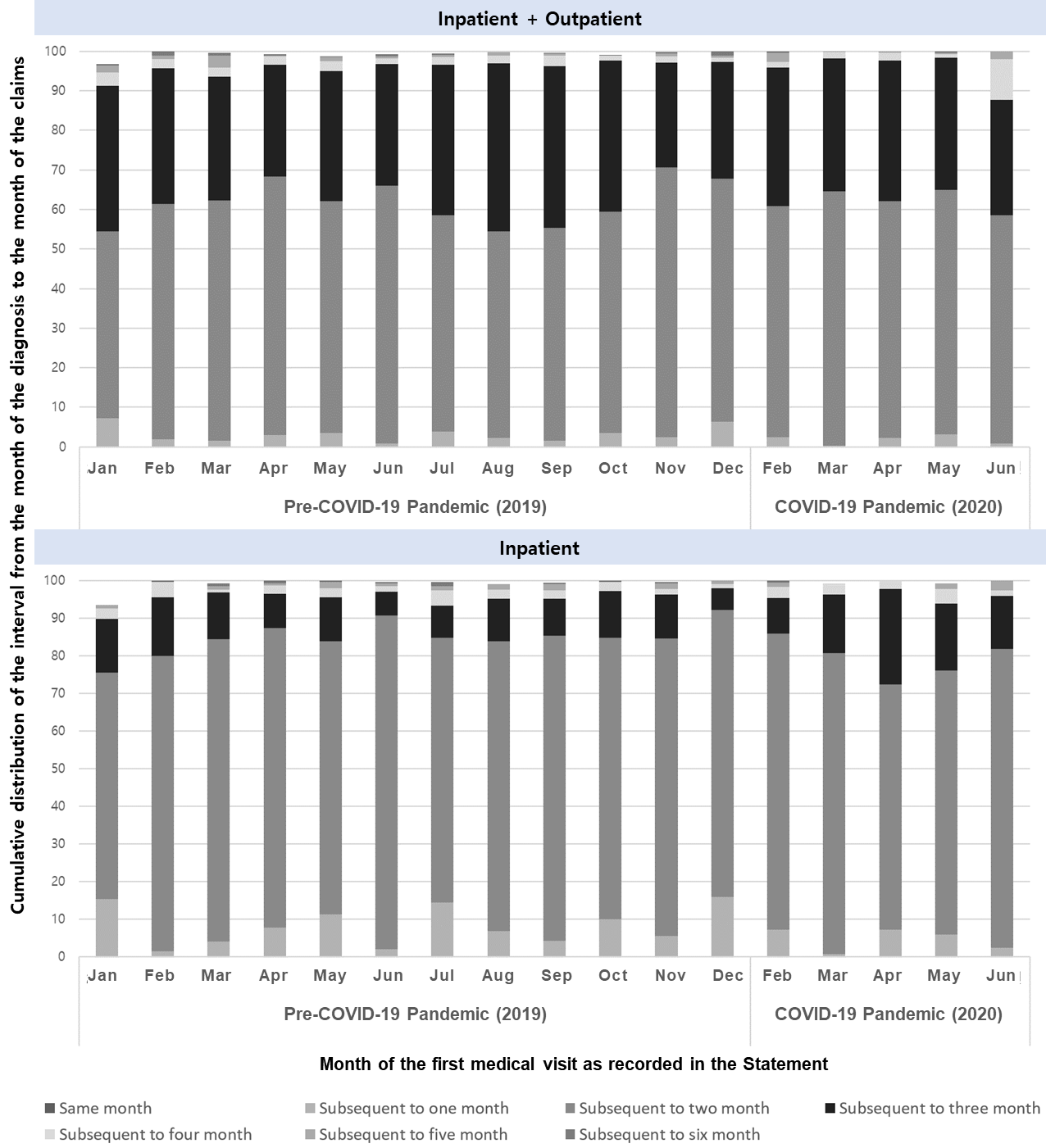


Supplemental Figure S8. Distribution of the interval between diagnosis month of myocarditis and data accrual month.


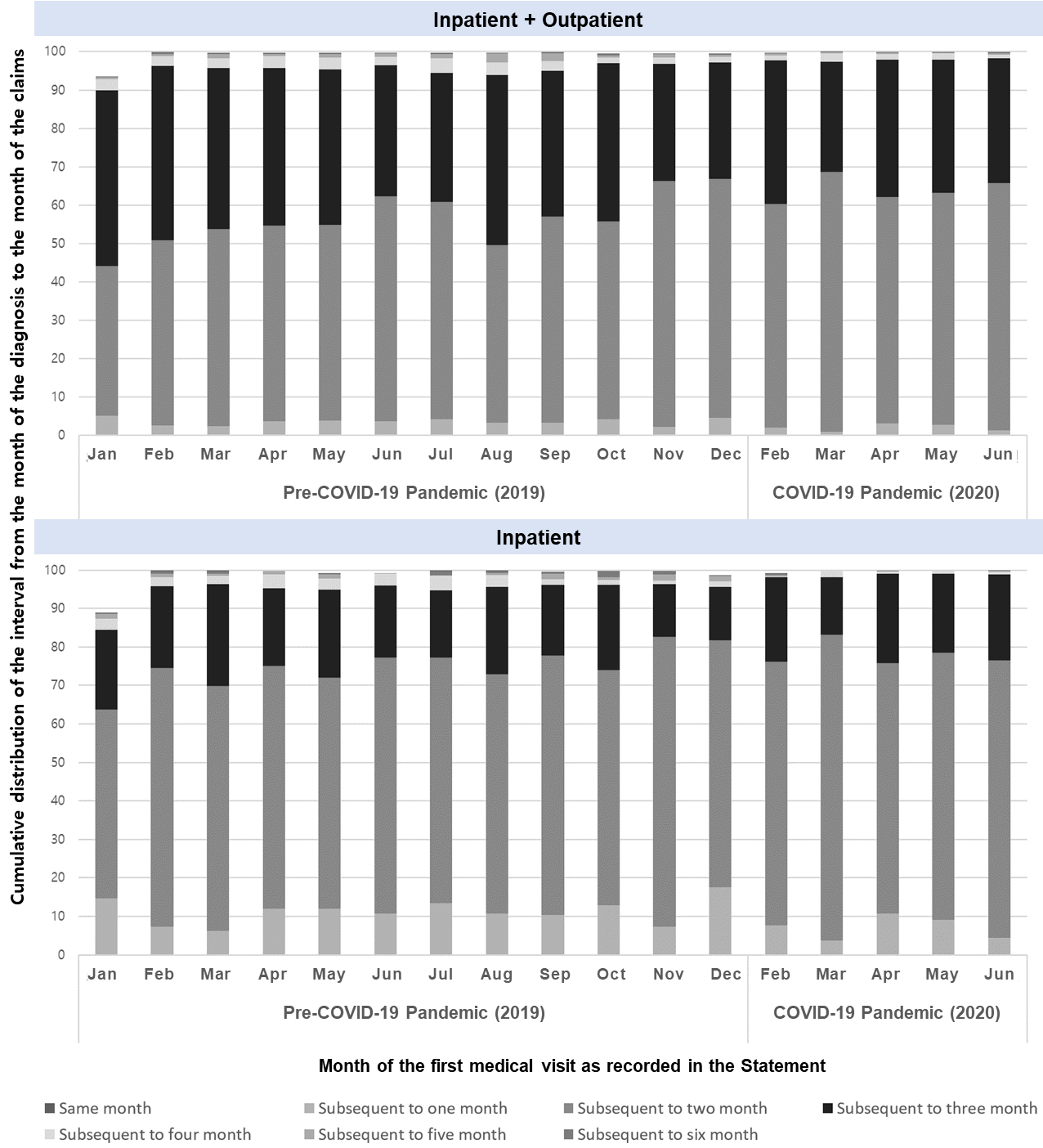


Supplemental Figure S9. Distribution of the interval between diagnosis month of anaphylaxis and data accrual month.


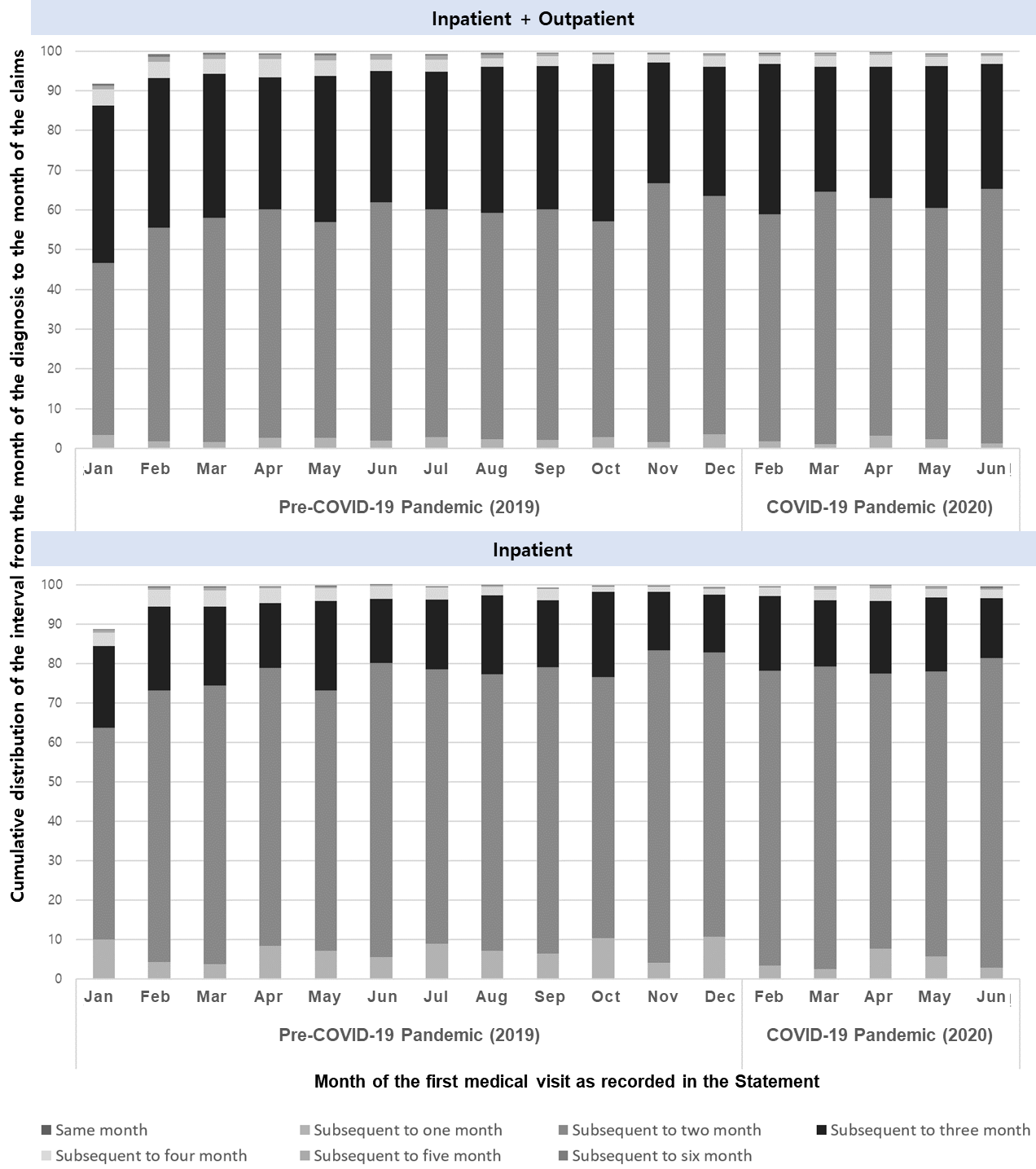


Supplemental Figure S10. Distribution of the interval between diagnosis month of colonic diverticulitis and data accrual month.


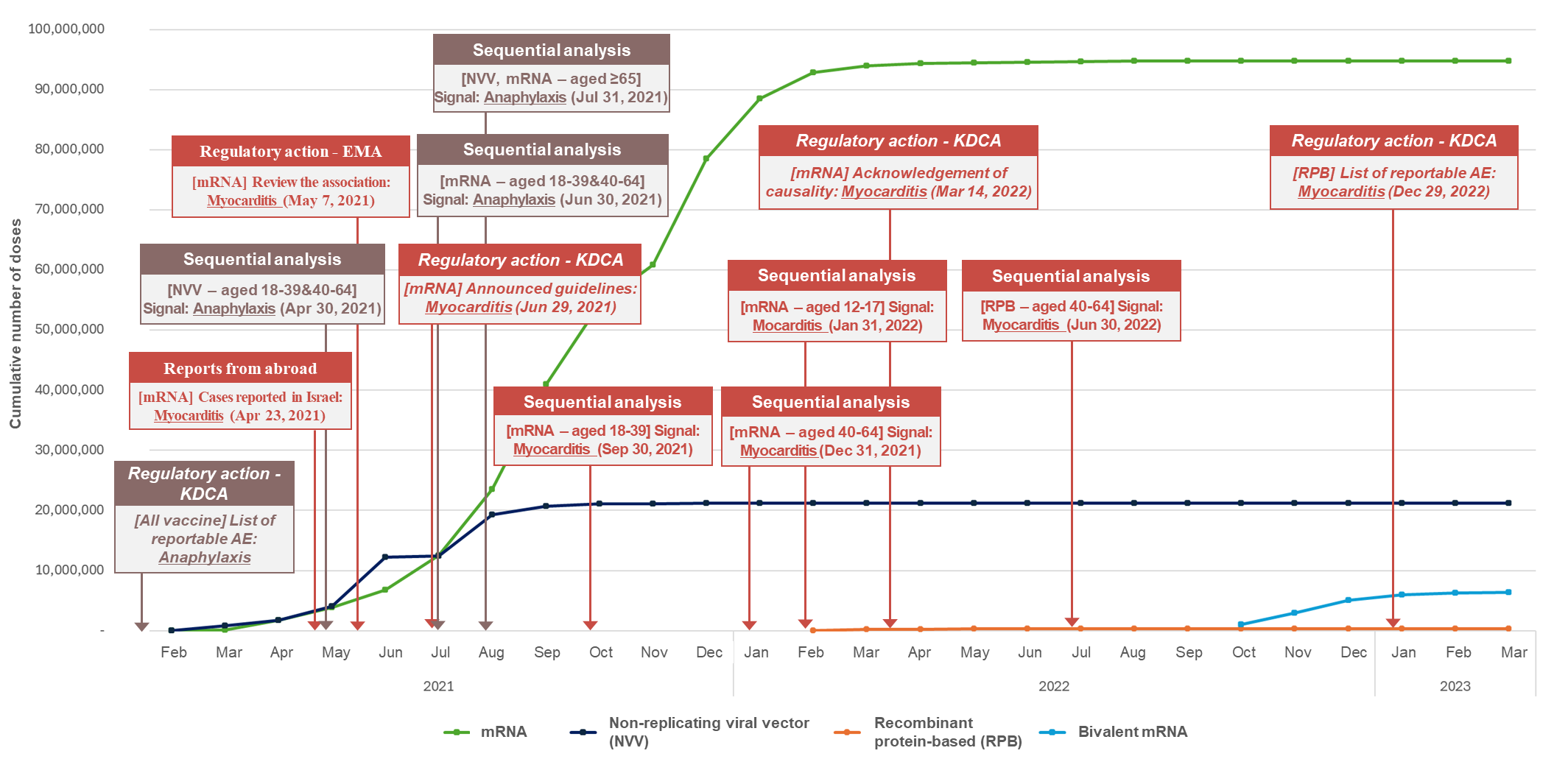


Supplemental Figure S11. Timing of meeting statistical signal threshold based on sequential testing and regulatory actions for adverse events related to COVID-19 vaccines.
